# Overcoming the Crystallization Bottleneck: A Family of Gigantic Inorganic {Pd_*x*_}^L^ (*x=*84, 72) Palladium Macrocycles Discovered using Solution Techniques

**DOI:** 10.1002/anie.201606005

**Published:** 2016-09-16

**Authors:** Lorna G. Christie, Andrew J. Surman, Rachel A. Scullion, Feng Xu, De‐Liang Long, Leroy Cronin

**Affiliations:** ^1^ WEST Chem, School of ChemistryUniversity of GlasgowUniversity AvenueGlasgowG12 8QQUK

**Keywords:** ion mobility, palladium, polyoxopalladates, self-assembly, size exclusion

## Abstract

The **{Pd_84_}^Ac^** wheel, initially discovered serendipitously, is the only reported giant palladium macrocycle—a unique structure that spontaneously assembles from small building blocks. Analogues of this structure are elusive. A new modular route to **{Pd_84_}^Ac^** is described, allowing incorporation of other ligands, and a new screening approach to cluster discovery. Structural assignments were made of new species from solution experiments, overcoming the need for crystallographic analysis. As a result, two new palladium macrocycles were discovered: a structural analogue of the existing **{Pd_84_}^Ac^** wheel with glycolate ligands, **{Pd_84_}^Gly^**, and the next in a magic number series for this cluster family—a new **{Pd_72_}^Prop^** wheel decorated with propionate ligands. These findings confirm predictions of a magic number rule for the family of {Pd_x_} macrocycles. Furthermore, structures with variable fractions of functional ligands were obtained. Together these discoveries establish palladium clusters as a new class of tunable nanostructures. In facilitating the discovery of species that would not have been discovered by orthodox crystallization approaches, this work also demonstrates the value of solution‐based screening and characterization in cluster chemistry, as a means to decouple cluster formation, discovery, and isolation.

One of the most striking phenomena in inorganic chemistry is the formation of nanoscale inorganic macrocycles—whereby large structures can self‐assemble from simple building blocks—and their unusual properties (such as single molecule magnet (SMM) behavior,^1^ with potential applications in quantum computing).^2^ One large family of nanoscale metal oxide macrocycles have been known for some time: Mo‐blue wheels,^3^ which can form a huge variety of different sized supramolecular architectures.^4^ Christou et al. have reported another family based on manganese: first {Mn_84_}, and more recently {Mn_70_}, both of which behave as SMMs.^5^, ^6^ Our group recently reported a new nanoscale metal oxide macrocycle, **{Pd_84_}^Ac^** (Na_56_H_14_[Pd_84_O_42_(CH_3_CO_2_)_28_(PO_4_)_42_]), the largest known polyoxopalladate (POPd) to date,7a–11 and potentially the parent of a third family of macrocycles. However, efforts to produce analogues have been in vain, illustrating that synthesis of these structures is not straightforward and that the underlying principles are not well‐understood. Only with understanding can we make the transition from discovery to sophisticated control over macrocycle design and synthesis, and finally, exploitation of their properties.

Herein, we describe our rational efforts to build a family of POPd wheels. The discovery of **{Pd_84_}^Ac^** was serendipitous, using single crystal X‐ray crystallography to identify crystals formed after stirring Pd(OAc)_2_ in phosphate buffer (Scheme 1 a).^11^, ^12^ Most large inorganic nanostructures are discovered in a similar way, requiring reactions not just to proceed, but to also produce pure diffraction‐quality crystals. Where no such crystals are produced, no structure is determined and no compound is discovered; that is, a “crystallization bottleneck”. Herein, we demonstrate how new metal oxide macrocycles can be discovered without the need for diffraction‐quality crystals. Instead, we exploit solution sizing techniques^12^ to screen reactions for the presence of new nanoscale species, even where no crystals are observed. Data suggesting new species (“leads”), discovered either by screening or crystallization, are then structurally characterized from crude solution. We could then focus further efforts on isolation of the compounds by crystallization. As a result of these new approaches, we report the discovery of two new members of the POPd wheel family. We also demonstrate the formation of a tunable range of wheels with mixed acetate/glycolate ligand functionality.

**Scheme 1:**
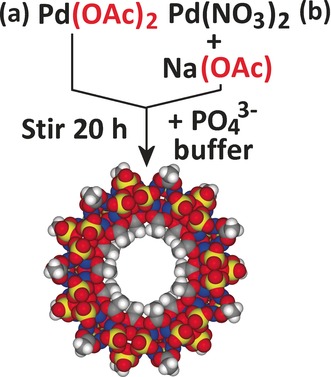
Two synthetic routes to **{Pd_84_}^Ac^** using different palladium sources: a) the original route, using palladium acetate; b) a new route, using palladium nitrate.

As outlined above, the original synthesis of **{Pd_84_}^Ac^** uses Pd(OAc)_2_ as a starting material (Scheme 1 a). To synthesize wheels with other carboxylate ligands, the first challenge we faced was to design a new modular synthetic route, separating the carboxylate source from the palladium source, allowing ligands to be varied independently. This was accomplished using palladium nitrate as a palladium(II) source, and sodium acetate as a separate carboxylate source, which was readily substituted for other sodium carboxylate salts (Scheme 1 b). This new route yielded **{Pd_84_}^Ac^** crystals in similar yields to the previous approach (see the Supporting Information for the method and characterization details). Having validated this alternative synthetic route, a series of experiments were attempted substituting different carboxylate ligands (as sodium salts). Of these, no reaction produced crystals of sufficient quality for structural determination by X‐ray diffraction (XRD) and only one reaction—substituting propionate for acetate—produced any crystalline material at all.

Despite a lack of crystalline products suitable for XRD, it was clear from observing color changes and performing gel electrophoresis^12^ that palladate cluster products were being formed in the reaction solutions. In the course of our previous studies on the formation of **{Pd_84_}^Ac^**, we developed a size exclusion chromatography (SEC) method in which POPd clusters of different sizes are resolved; in so doing we were able to show that macrocycles are present in reactions even when no crystals are formed.^12^ Knowing there to be products in the reaction mixtures, we screened crude solutions of reactions incorporating a wide variety of carboxylate ligands for the synthesis of large species. That is, instead of setting up many reactions and waiting for serendipitous crystallization, our new approach screened for the formation of large species as leads to be further characterized. To do this, we compared the relative amounts of large species observed by comparing the relative integrals of a screening range (from 12–17.3 min), corresponding to where **{Pd_84_}^Ac^** or other large species were expected to elute (in SEC, larger species elute earlier).

The ligands to be screened were chosen to incorporate carboxylate moieties (including some di‐ and trivalent examples), along with a range of other functional groups (amine, alcohol, aromatic, and fluorine). Other than substituting acetate for the new ligand, the reaction procedure was the same as the new modular **{Pd_84_}^Ac^** reaction (scaled down, to preserve the expensive palladium nitrate starting material). Since **{Pd_84_}^Ac^** was previously found to form in solution over six days (persisting for weeks), nine days were allowed for macrocycle formation before screening. The results can be seen in Figure 1 b, expressed as relative amounts of material observed to elute in the range corresponding to large clusters. Under these conditions, the reaction using glycolate (ligand “O”) stands out as yielding considerable amounts of large species in solution. The chromatogram for this reaction mixture consisted of a large peak with a very similar retention time to that of **{Pd_84_}^Ac^** (Supporting Information, Figure S1). This is consistent with the formation of a **{Pd_84_}** macrocycle surrounded by glycolate ligands in place of acetate ligands (denoted as **{Pd_84_}^Gly^**). Furthermore, we see no distinct peaks eluting beyond 16.7 min, suggesting no significant amounts of smaller clusters are present (for example, {Pd_15_} or {Pd_10_}). We note that no crystals were formed from these screening reactions and that any “leads” would not have been identified using a more orthodox approach.

**Figure 1:**
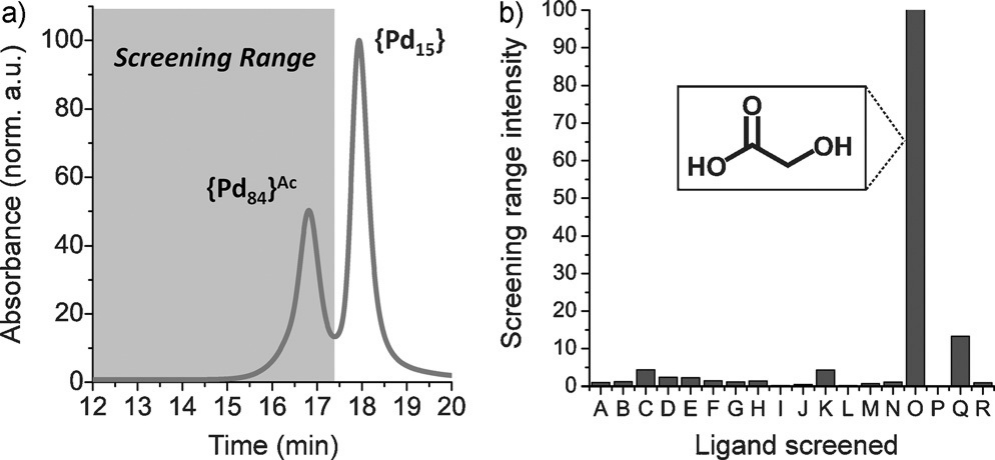
a) SEC chromatogram of **{Pd_84_}^Ac^** (large macrocycle) and {Pd_15_} (smaller cluster), demonstrating the screening range in which large POPd wheels might be expected to elute (absorbance at 350 nm). b) Results of screening a selection of carboxylate ligands, where the normalized screening range (12.0–17.3 mins) intensities represent the relative amounts of large POPd species present in the reaction solution. Ligand identifiers: A, benzoate; B, 3‐hydroxypropanoate; C, squarate; D, alanine; E, oxalate; F, ethylenediaminetetraacetate; G, citrate; H, 1,2,3,4‐cyclobutanetetracarboxylate; I, 1,4‐cyclohexanedicarboxylate; J, nitrilotriacetate; K, isonicotinate; L, isophthalate; M, croconate; N, itaconate; O, glycolate; P, trifluoroacetate; Q, malonate; R, 2‐pyridinecarboxylate.

Having obtained strong solution evidence of new large palladate clusters with propionate and glycolate ligands, but no diffraction‐quality crystals, our attention turned to characterization of these structures. We have been developing electrospray ionization ion mobility mass spectrometry (ESI‐IMS‐MS) as a tool for cluster discovery, characterization, and structural assignment.^13^, ^14^, ^15^ This technique is valuable as it gives information on size and shape—as collision cross‐section (CCS_He_)—as well as composition, *m*/*z*. ESI‐IMS‐MS was carried out on an aqueous solution of pure **{Pd_84_}^Ac^** crystals (Figure 2 a), a sample of the glycolate reaction mixture from the ligand screening (Figure 2 b, reaction “O” from ligand screening), and a solution of the crystalline material obtained from the substitution of propionate as a ligand (Figure 2 c), all desalted to avoid ionization suppression (Supporting Information, Section 2). As can be observed in Figure 2, a single series of broad peak envelopes is observed in each spectrum, showing that only one major structure is present in each case. The spectrum yielded by **{Pd_84_}^Ac^** (Figure 2 a) is consistent with previous ESI‐MS observations of a series of broad peaks corresponding to a manifold of [Pd_84_O_42_(CH_3_CO_2_)_28_(PO_4_)_42_] (Na)_*x*_(H)_*y*_(H_2_O)_*z*_ ions (*x*, *y*, and *z* vary, hence broad peaks) and reveals a CCS_He_ of 1070 Å. The product of the glycolate substitution reaction mixture yields a spectrum (Figure 2 b) that is strikingly similar to that of **{Pd_84_}^Ac^** in apparent mass, charge distribution, and CCS_He_. Taken together, this data allows us to assign it as **{Pd_84_}^Gly^**, a new **{Pd_84_}^Ac^** structural analogue. It might be expected that ligand substitution with propionate would also yield an analogous **{Pd_84_}** macrocycle. However, ESI‐IMS‐MS shows this not to be the case (Figure 2 c); the main product observed is smaller in mass and size (CCS_He_ of 929 Å, compared to 1070 Å for **{Pd_84_}^Ac^**) corresponding to a smaller, but nonetheless well‐defined, **{Pd_72_}^Prop^** cluster. Having converted these “leads” into “hits”, and assigned structures using solution methods, we then aimed to produce diffraction‐quality crystals from these two reactions to validate our structural assignments.

**Figure 2:**
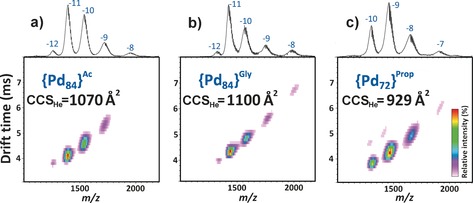
ESI‐IMS‐MS spectra of a) **{Pd_84_}^Ac^**, b) **{Pd_84_}^Gly^**, and c) **{Pd_72_}^Prop^**, showing the respective collision cross‐sections (Ω) in angstroms squared [Å^2^]. A 5 % noise threshold was applied for clarity.

Efforts to obtain diffraction‐quality crystals of the new **{Pd_72_}^Prop^** met with success more readily. Upon XRD analysis, the crystallographic data reveals a new macrocycle with 72 palladium centers, with a general formula Na_60_[Pd_72_O_36_(C_2_H_5_CO_2_)_24_(PO_4_)_36_]⋅≈200 H_2_O, denoted **{Pd_72_}^Prop^**. The wheel has six‐fold symmetry, differing from the seven‐fold symmetry of the original **{Pd_84_}^Ac^** wheel (Figure 3). We note that this observation is consistent with our previous prediction, and that on the basis of the discovery of **{Pd_84_}^Ac^** and the existence of {Mn_84_}, the next clusters in each series would be {Pd_72_} and {Mn_70_}, respectively.^6^, ^11^ Structurally, we can observe the same {Pd_6_} subunits (Supporting Information, Figure S20) as those in **{Pd_84_}^Ac^**; the smaller size and six‐fold symmetry of this new wheel arise from incorporating twelve of these {Pd_6_} units, as opposed to fourteen in **{Pd_84_}^Ac^**. **{Pd_72_}^Prop^** has a slightly smaller overall diameter (ca. 3.1 nm compared with ca. 3.4 nm for **{Pd_84_}^Ac^**, measured across the outermost ligands) and a significantly smaller inner cavity size compared to **{Pd_84_}^Ac^** because of the accommodation of longer and bulkier propionate ligands. During the **{Pd_72_}^Prop^** synthesis, a new, smaller cluster also forms as a side product, which was identified as a neutral decanuclear cluster [Pd_10_O_4_(C_2_H_5_CO_2_)_12_]⋅(CH_3_CN)_3_ crystallographically, denoted **{Pd_10_}^Prop^** (Supporting Information, Figure S19). This is analogous to the {Pd_10_}^Ac^ cluster reported previously.^12^ ^31^P NMR analysis of a solution of **{Pd_72_}^Prop^** crystals further confirms its structural similarity to **{Pd_84_}^Ac^**, showing that the new macrocycle also comprises two inequivalent phosphorous environments.^11^ Interestingly, ^31^P NMR also revealed minor peaks, up‐field of the main peaks, which grow over time (see the Supporting Information for the difference over 24 h); these are thought to correspond to a degradation product of **{Pd_72_}^Prop^**, demonstrating limited stability in pure water. This degradation was also observable in SEC (over a 20 h period; Supporting Information, Section 7) and by ESI‐IMS‐MS (we attribute the faint second series of peaks at longer drift times to the same degradation product, Figure 2 c).

**Figure 3:**
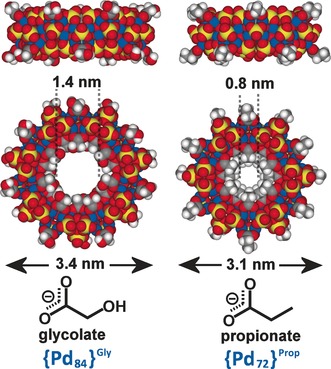
Space filling representation of the two new crystal structures of **{Pd_84_}^Gly^** (left) and **{Pd_72_}^Prop^** (right), with the corresponding overall and inner cavity diameters. Key: Pd, blue; O, red; P, yellow; C, grey. Hydrogen atoms omitted for clarity.

Efforts to isolate diffraction quality crystals of **{Pd_84_}^Gly^** were met with less initial success, attributed to the cluster being considerably more hydrophilic than **{Pd_84_}^Ac^**; we only persevered because of the strong SEC and ESI‐IMS‐MS evidence that a new **{Pd_84_}^Gly^** species was present in solution. After many attempts, rhomboid shaped crystals appeared from a concentrated mother liquor and XRD confirmed this new structure to be **{Pd_84_}^Gly^**, a macrocycle of general formula Na_56_H_14_[Pd_84_O_42_(CH_4_CO_3_)_28_(PO_4_)_42_]⋅≈200H_2_O and direct ligand‐substituted analogue of **{Pd_84_}^Ac^**. **{Pd_84_}^Gly^** exhibits the same {Pd_6_} building units as both **{Pd_84_}^Ac^** and **{Pd_72_}^Prop^** (Supporting Information, Figure S21), with identical bonding between the units. **{Pd_84_}^Gly^** contains fourteen of the {Pd_6_} building units, giving rise to the same seven‐fold symmetry seen in **{Pd_84_}^Ac^**.

The only difference in the reactions producing **{Pd_72_}^Prop^** and **{Pd_84_}^Ac^** is the identity of the ligand added; both produce differently sized macrocycles. This suggests that we have not only established a method to produce analogues of the **{Pd_84_}^Ac^** archetype, but also established ligand identity as a means of controlling macrocycle size. Where two ligands lead to the same macrocyclic size, however, they may be mixed. This was shown in proof of concept experiments using a mixture of acetate and glycolate ligands at varying ratios, (1:9, 2:8, 3:7, and so forth; Supporting Information, Section 9). As the mole fraction of glycolate ligand was increased, the overall mass of the product observed increased gradually (Supporting Information, Figures S28 and S29). This is a strong indication that we obtain mixed functionality wheels, rather than two homo‐ligand wheels. Glycolate ligands bear alcohol functional groups. The ability to tune the amount of these functional ligands on a ring holds great synthetic promise, both as a means to tune macrocycle properties (for example, solubility), and to incorporate moieties amenable to further functionalization, either before or after macrocycle synthesis.

In conclusion, we have reported a new modular synthetic route to produce giant POPd macrocycles, allowing us to independently vary the carboxylate ligand. This has facilitated expansion of the family of POPd wheels by the synthesis of two new structures, **{Pd_84_}^Gly^** and **{Pd_72_}^Prop^**. Furthermore, we have demonstrated the availability of a range of tunable mixed‐ligand structures, and that the macrocycle size can be controlled by choice of ligand. Crucially, the exploration of the clusters in solution using SEC and ESI‐IMS‐MS facilitated screening for new species in reaction mixtures without producing diffraction‐quality crystals—the “crystallization bottleneck” in the discovery workflow—thereby decoupling synthesis and isolation from discovery. The sparse distribution of “hits” available over the parameter space screened—that is, the fact that only a few ligands produced POPd macrocycles—demonstrates the advantage of screening over traditional discovery, where optimizing crystallization for each individual reaction can take months. The conditions included in this screen have been limited, resembling those producing the original **{Pd_84_}^Ac^** structure. We expect that by investigating a wider range of conditions using the same approach, we will add many more members to the palladium wheel family. Further to this, the SEC screening approach can be more readily automated than conventional techniques. We have found some validation for the “magic number” rule we previously predicted^11^ for this emerging family of {Pd_*x*_} (Figure 4; also seen in recent reports on {Mn_*x*_} macrocycles). We hope that by expanding the range of macrocycles available for study we will further reveal links between symmetry, building blocks, and structure in the assembly of gigantic inorganic systems, thereby aiding the transition from discovery to design.

**Figure 4:**
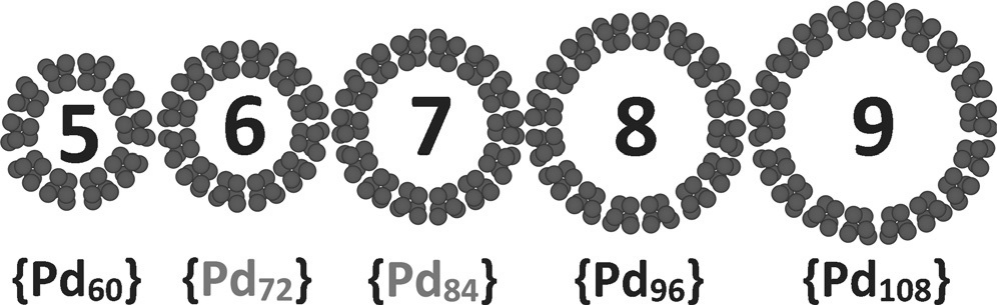
Metal‐only filling representation of the building block numbers in {Pd_*x*_} from 5×12=60 up to 9×12=108. The two new crystal structures of **{Pd_84_}^Gly^** (6) and **{Pd_72_}^Prop^** (7) are also shown. Clusters {Pd_60_}, {Pd_96_}, and {Pd_108_} are hypothetical architectures consistent with the magic numbers suggested herein.

## Experimental Section

Full synthesis, analytical, and structural details are provided in the Supporting Information, including experimental details and characterization for **{Pd_84_}^Gly^** and **{Pd_72_}^Prop^** (SEC‐HPLC, ESI‐IMS‐MS, and detailed assignments). CCDC 1484605 http://www.ccdc.cam.ac.uk/cgi‐bin/catreq.cgi (**{Pd_84_}^Gly^**), 1484604 http://www.ccdc.cam.ac.uk/cgi‐bin/catreq.cgi (**{Pd_72_}^Prop^**), and 1484603 http://www.ccdc.cam.ac.uk/cgi‐bin/catreq.cgi (**{Pd_10_}^Prop^**) contain the supplementary crystallographic data for this paper. These data can be obtained free of charge from The Cambridge Crystallographic Data Centre.

## Supporting information

As a service to our authors and readers, this journal provides supporting information supplied by the authors. Such materials are peer reviewed and may be re‐organized for online delivery, but are not copy‐edited or typeset. Technical support issues arising from supporting information (other than missing files) should be addressed to the authors.

Supplementary
