# Supplementary material for: Overcoming the Crystallization Bottleneck: A Family of Gigantic Inorganic {Pdx}L (x=84, 72) Palladium Macrocycles Discovered using Solution Techniques
Source: Angew Chem Int Ed Engl. 2016 Sep 16;55(41):12741–5. doi: 10.1002/anie.201606005 (PMC5113701; doi:10.1002/anie.201606005)
Supplement: Supplementary file 1 — Supplementary [file ANIE-55-12741-s001.pdf]

Angewandte Chemie
Eine Zeitschrift der Gesellschaft Deutscher Chemiker

Supporting Information

# Overcoming the Crystallization Bottleneck: A Family of Gigantic Inorganic $\{Pd_x\}^L$ ($x = 84, 72$) Palladium Macrocycles Discovered using Solution Techniques

*Lorna G. Christie[+], Andrew J. Surman[+], Rachel A. Scullion, Feng Xu, De-Liang Long, and Leroy Cronin**

anie_201606005_sm_miscellaneous_information.pdf

# Supporting information:

**Index** **Page**


1. Materials and methods S2
2. De-salting using MicroBioSpin™ 6 columns S4
3. Synthetic procedures S4
4. SEC screening and chromatograms S7
5. Crystallographic data S14
6. $^{31}P$ NMR S19
7. Degredation of $\{Pd_{72}\}^{Prop}$ in SEC S20
8. ESI-IMS-MS Studies S21
9. Mixed ligand mass spectrometry S29
9. References S31

## (1) Materials and methods

All chemicals and solvents were used as purchased without further purification.

**SEC:** Isocratic SEC analyses were performed on an Agilent 1100 Series (Agilent Technologies) equipped with a vacuum degasser, a binary pump (G1312A), and a diode array detector (DAD) (G1315A). 5 $\mu$L of the samples (1:4 dilution in water) were injected on an OHPak SB-804m (Shodex) 10µm, 8.0 x 300 mm column and eluted at 0.5 mL/min of 0.05 M sodium acetate buffer (pH = 5.5) for separation measurements. Elution was detected by UV (λ = 350 nm) and samples were run for 30 minutes. The data recorded were processed using Bruker Hystar 3.2 software (Bruker Daltonics). All SEC runs included the standard 1:1 mixture of $\{Pd_{84}\}$ and $\{Pd_{15}\}$, to ensure consistent results. Screening data was output as chromatograms (.csv files; batch output automated using AutoHotKey[1]) and processed (integrated, normalised) in an automated manner using a custom script written in R.[2]

**Microanalysis:** Carbon, nitrogen and hydrogen content were determined by the microanalysis services within the School of Chemistry, University of Glasgow using an EA 1110 CHNS, CE-440 Elemental Analyzer.

**$^{31}$P Nuclear Magnetic Resonance Spectroscopy:** $^{31}$P NMR spectroscopy were recorded on a Bruker DPX 500 spectrometer using the solvent signal as internal standard. All samples were prepared by dissolving the samples in $D_2O$, or desalting with spin columns as detailed in SI section (2), followed by addition of $D_2O$.

**Single Crystal X-Ray Diffraction:** Single-crystal x-ray diffraction experiments were performed on either (a) a Bruker APEXII Quasar CCD area detector diffractometer using graphite-monochromated Mo Kα radiation (λ = 0.71073 Å) equipped with an Oxford Cryosystems open-flow cryostat operating at 150 K; (b) dectris-CrysAlisPro-abstract goniometer imported dectris, mounted on Beamline I19 at the UK Diamond Light Source (λ = 0.6889 Å) at 100 K; (c) an Atlas CCD area detector on an Oxford diffraction Gemini A Ultra diffractometer using graphite-monochromated Mo Kα radiation (λ = 0.71073 Å) and equipped with an Oxford Cryosystems open-flow cryostat operating at 150 K; The structures were solved by direct methods using SHELXS97[3] or SHELXT[4] and refined by full-matrix least squares on F2 using SHELXL2014.[5]

It is noted that due to the high hexagonal symmetry and heavy disorder observable in the packing of the clusters, the quality of the dataset collected for the $\{Pd_{72}\}^{Prop}$ structure is lower than for a high resolution small molecule structure. Despit the relatively high R1 of 0.17, the refined model of the $\{Pd_{72}\}^{Prop}$ structure clearly allows us to elucidate all the main features of the cluster and the structure also shows the $\{Pd_{6}\}$ building unit, similar to that of the $\{Pd_{84}\}$ clusters. Based on this similarity, the

hexagonal symmetry and elemental analysis, the composition of the $\{Pd_{72}\}^{Prop}$ cluster compound can be determined beyond doubt. Despite the disorder limitations, it is clear that the $\{Pd_{72}\}^{Prop}$ crystal structure is of sufficient quality to confirm our structural characterisation of the new cluster as a $\{Pd_{72}\}^{Prop}$ macrocycle (having already assigned this structure by other means).

**(2) De-salting using MicroBioSpin™ 6 columns**

To prevent ion suppression resulting from the excess of salt/buffer present in polyoxopalladate reaction mixtures (or resulting from partial breakdown in the case of solutions of pure compounds in water), we have adopted a desalting approach using MicroBioSpin™ 6 columns[6]. The columns are packed with special grades of Bio-Gel® P polyacrylamide P-6 gel, a size exclusion resin which allows larger species such as $\mathbf{\{Pd_{84}\}^{Ac}}$ to pass through, and retaining salts/ smaller species. Nominal cut-off is around 6 kDa, although this is based on the size of globular proteins; our observations suggest that for rigid oxide clusters, this cut-off lies around the size of $\mathbf{\{Pd_{15}\}^{Ac}}$, the concentration of which is reduced by around 50% with each desalting 'pass'.

For analysis, the general procedure is to desalt reaction solutions twice using MicroBioSpin 6 columns. The columns are stored in TRIS buffer which is removed by flushing with 2 x aliquots of 500 µL $NH_4OAc$, then 3 x 500 µL HPLC $H_2O$. The washings are passed through the columns by centrifuging for 2 minutes at 3700 rpm. 75 µL of the sample is then loaded for desalting, and centrifuged for 3 minutes at 3700 rpm.

## (3) Synthetic Procedures

*Synthesis of* {**$Pd_{84}$**}$^{Ac}$

**Method 1:** (previously published) - $Pd(OAc)_2$ (0.56 g, 2.5 mmol) was added to $NaH_2PO_4$ / $Na_2HPO_4$ solution (22 ml, 0.15 M) at a pH of 6.9. The suspension was left stirring rigorously for 20 hours. The dark brown solution was centrifuged and filtered to remove a small amount of precipitate. The pH of the filtrate was then checked, and, if necessary, a few drops of 1M NaOH were added to adjust the pH to above 4.5. Evaporation of the filtrate from an open beaker led to the formation of red/brown needle-like crystals after a few days. The crystals were collected by filtration and air-dried. Yield: ca. 15 - 20% (based on Pd). For further information see Xu *et al. Proc. Natl. Acad. Sci. USA* **2012**, *109*, 11609-11612.

**Method 2:** $Pd(NO_3)_2$ (0.67 g, 3.0 mmol) and Na(OAc) (was added to $NaH_2PO_4$-$Na_2HPO_4$ solution (22 ml, 0.15 M) at a pH of 6.9. The suspension was left stirring rigorously for 20 hours. The dark brown solution was centrifuged and filtered to remove a small amount of precipitate. The pH of the filtrate was then checked, and, if necessary, a few drops of 1M NaOH were added to adjust the pH to above 4.5. Evaporation of the filtrate from an open beaker led to the formation of red/brown needle-like crystals after a few days. The crystals were collected by filtration and air-dried. Yield: ca. 3% (based on Pd). $^{31}P$ NMR (500 MHz, $D_2O$) δ 21.77, 13.17 ppm

*Synthesis of* {**$Pd_{72}$-propionate**} – $Pd(NO_3)_2$ (0.67 g, 3 mmol) and $Na(C_3H_5O_2)$ (0.48 g, 5 mmol) was dissolved in $NaH_2PO_4$-$Na_2HPO_4$ solution (30 ml, 0.15 M) at a pH of 6.9. The solution was left stirring rigorously for 20 hours. The resulting dark brown solution was centrifuged and filtered to remove a small amount of precipitate. The pH of the filtrate was then checked (normally in range 4.7-5.0) Evaporation of the filtrate from an open beaker led to the formation of red/brown needle-like crystals after a few days. Yield: ca. 2%(based on Pd). $^{31}P$ NMR (500 MHz, $D_2O$) δ 22.25, 13.47 ppm. The crystals were collected by filtration and dried in a desiccator for one week prior to analysis. TGA analysis showed a 6.7% mass loss up to 200 °C. This corresponds to water loss so elemental analysis was calculated for the dried formula. Calculated $C_{72}H_{280}Na_{60}O_{288}P_{36}Pd_{72}$: C 5.45%, H 1.52% Na 8.69%, P 7.02%, Pd 48.28%; Found via ICP-OES: Na 8.36%, P 6.87%, Pd 42.0%; via CHN analysis; C 3.82%, H 1.73%. It is noted that in the CHN analysis we detected 0.66% Nitrogen. This indicates that there is some $Pd(NO_3)_2$ starting material still present, which is unsurprising considering the crystals form from a viscous mother liquor which may account for the variation between our expected and obtained values.  Attempts at washing and recrystallizing the material to obtain a purer sample for analysis have been unsuccessful.

*Synthesis of* {**$Pd_{10}$-propionate**} - $Pd(NO_3)_2$ (0.67 g, 3 mmol) and $Na(C_3H_5O_2)$ (0.48 g, 5 mmol) were dissolved in $NaH_2PO_4$-$Na_2HPO_4$ solution (22 ml, 0.15 M) at a pH of 6.9. The solution was left stirring rigorously for 20 hours. The resulting dark brown solution was centrifuged and the resulting precipitate forming at the bottom was dissolved in MeCN. Evaporation of the MeCN at room temperature from an open beaker led to the formation of orange/red block shaped crystals after one day. The crystals were collected by filtration and air-dried. Yield: ca. 10% (based on Pd). Elemental analysis calculated for $C_{40}H_{66}N_2O_{28}Pd_{10}$, C 23.02%, H 3.19%, N, 1.34%; Found C 21.96%, H 3.13%, N, 1.00%.

*Synthesis of* {**$Pd_{84}$-glycolate**} – $C_2H_4O_3$ (0.038 g, 0.5 mmol) and NaOH (0.02 g, 0.5 mmol) were dissolved in $NaH_2PO_4$-$Na_2HPO_4$ solution (22 ml, 0.15 M) at a pH of 6.9. To this solution was added $Pd(NO_3)_2$ (0.067 g, 0.3 mmol). The solution stirred vigorously for 20 hours. The resulting dark red solution was centrifuged and then filtered. The filtrate was evaporated from an open beaker for a minimum of 8 days before solution analysis. For further analysis, including mass spectrometry, the solution was typically desalted using MicroBioSpin™ 6 columns as deailed in section (2). Yield is currently unknown as a pure solid sample has not been obtained other than the few crystals grown on a small scale. Consequently, the formula has been determined solely by X-ray analysis.

***Synthesis for small scale screening reactions***

Two synthesis methods are listed because some of the ligand molecles screened were available as sodium salts and others were not. For the molecules not available as sodium salts, a stoichiometric amount of NaOH was added to the reaction.

**Method 1 - ligands available as sodium salts:** $Pd(NO_3)_2$ (0.067 g, 0.3 mmol) and Na(carboxylate) (0.5 mmol) were added to $NaH_2PO_4$-$Na_2HPO_4$ solution (2.2 mL, 0.15 M, pH 6.9). The reaction mixture was left stirring rigorously for 20 hours. The resulting solutions were filtered through syringe filters to remove any precipitate (some reactions had more precipitate than others). The solutions were left to evaporate from open vials for nine days before analysis using SEC.
The carboxylates reacted as sodium salts are as follows: sodium benzoate, sodium trifluoroacetate, sodium citrate, sodium oxalate, sodium malonate

**Method 2 - ligands available as carboxylic acids:** $Pd(NO_3)_2$ (0.067 g, 0.3 mmol), carboxylic acid (0.5 mmol) and a stoichiometric amount of NaOH (0.5 mmol for each carboxylate group present on the molecule) were added to $NaH_2PO_4$-$Na_2HPO_4$ solution (2.2 mL, 0.15 M, pH 6.9). The reaction mixture was left stirring rigorously for 20 hours. The resulting solutions were filtered through syringe filters to remove any precipitate. The solutions were left to evaporate from open vials for nine days before analysis using SEC. The carboxylates reacted using the carboxylic acid plus addition of NaOH

are as follows: 3-hydroxypropanoic acid, squaric acid, alanine, EDTA, 1,2,3,4-cyclobutanetetracarboxylic acid, 1,4-cyclohexanedicarboxylic acid, nitrilotriacetic acid, isonicotinic acid, isophthalic acid, croconoic acid, itaconic acid, glycolic acid, 2-pyridinecarboxylic acid.]

**Mixed ligand experiments**

$Pd(NO_3)_2$ (0.067 g, 0.3 mmol), sodium acetate, glycolic acid and a stoichiometric amount of NaOH (same number of moles as glycolic acid) were added to $NaH_2PO_4$-$Na_2HPO_4$ solution (2.2 mL, 0.15 M, pH 6.9). The ratio of acetate:glycole was varied from 0:10, 1:9, 2:8 etc, through to 10:0, ensuring the total amount of overall carboylate was kept as 5 mmol. The reaction mixtures were left stirring rigorously for 20 hours. The resulting solutions were filtered through syringe filters to remove any precipitate. The solutions were left to evaporate from open vials for nine days before analysis. Mass spectra for the mixed ligand reactions are shown in section (9).

## (4) SEC screening and chromatograms

Samples were prepared using the 'synthesis for small scale screening reactions' methods detailed in section **(3)**. Where the carboxylate salt was not commerically avaliable, it produced *in situ* using the corresponding acid and stoichiometric amount of NaOH (Method 2).

SEC chromatogtrams are shown below:

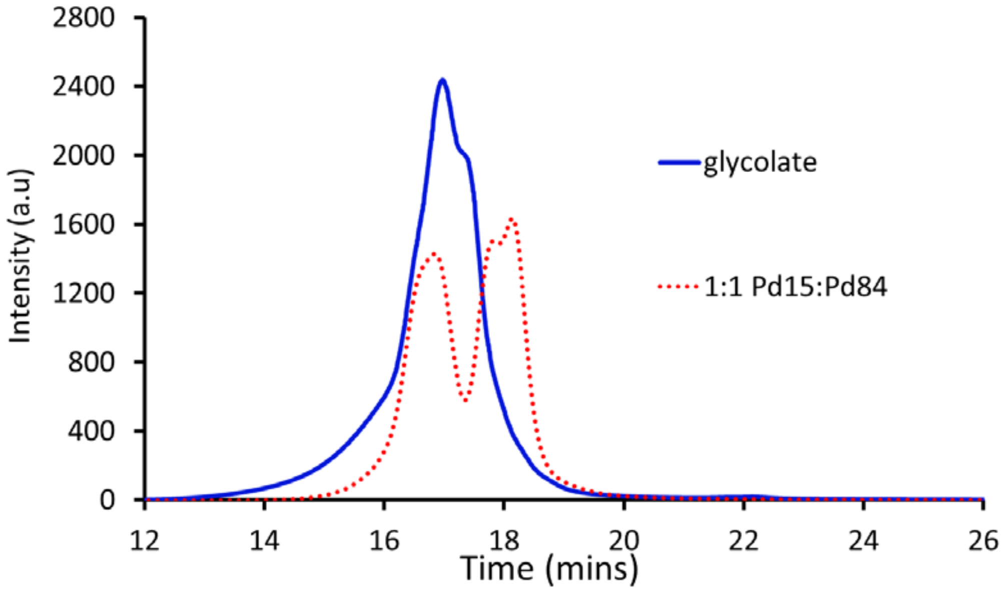


**Figure S1.** Chromatogram of reaction containing glycolic acid plotted with a 1:1 mix of $\{Pd_{84}\}^{Ac}$ and $\{Pd_{15}\}$ for comparison.

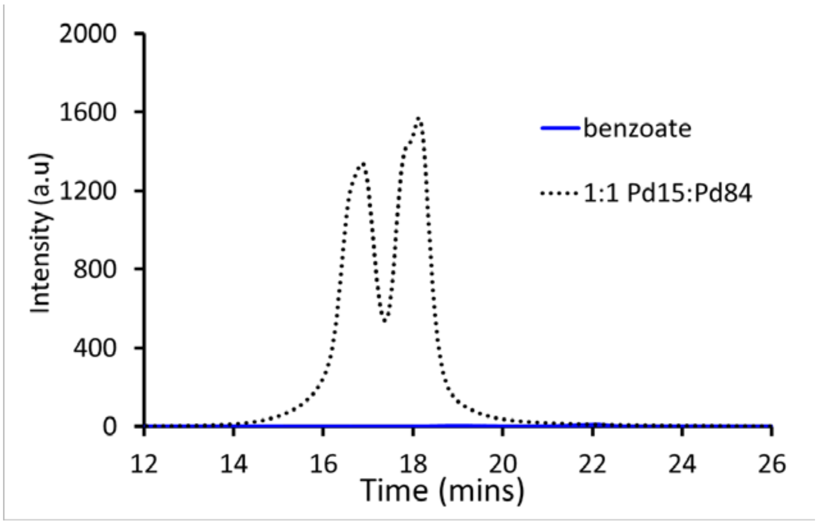


**Figure S2.** Chromatogram of reaction containing sodium benzoate (A from Figure 1b)

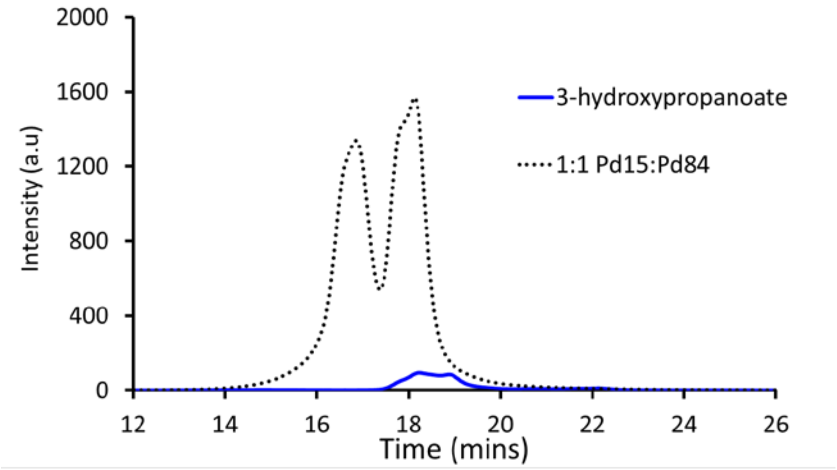


**Figure S3.** Chromatogram of reaction containing 3-hydroxypropanoic acid (B from Figure 1b)

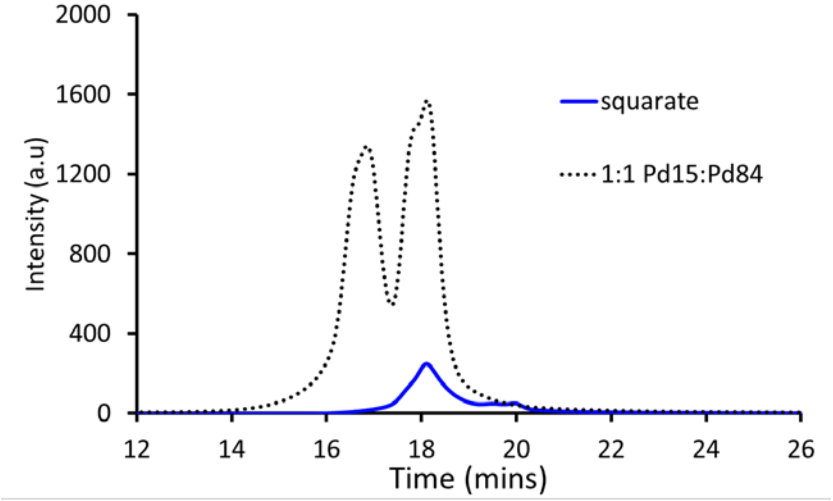


**Figure S4.** Chromatogram reaction containing squaric acid (C from Figure 1b)

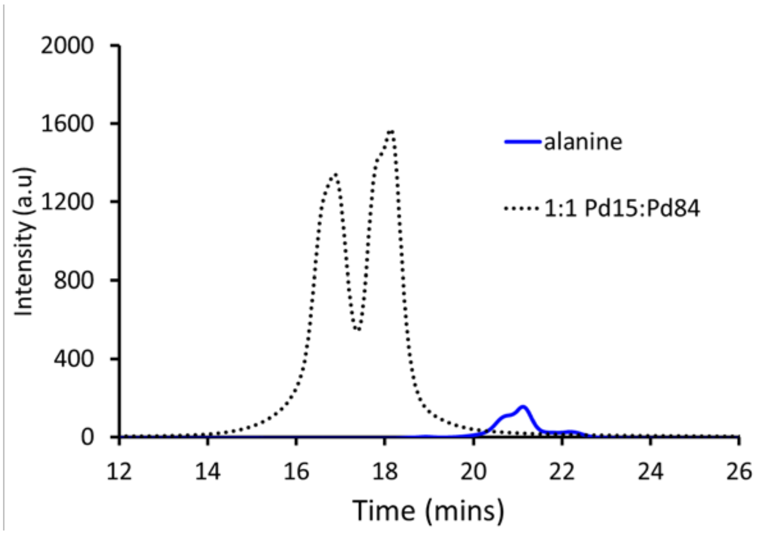


**Figure S5.** Chromatogram of reaction containing alanine (D from Figure 1b)

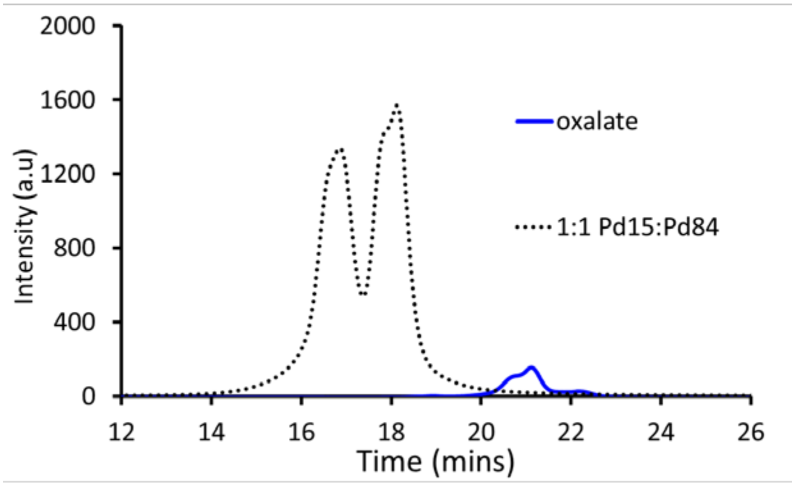


**Figure S6.** Chromatogram of reaction containing sodium oxalate (E from Figure 1b)

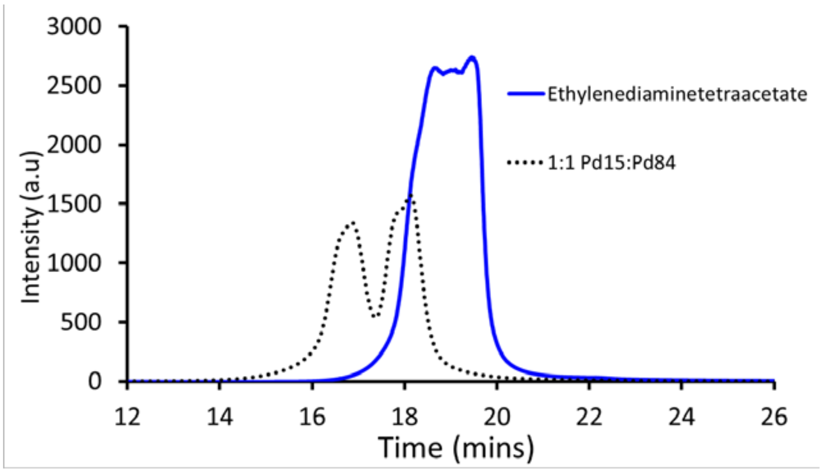


**Figure S7**. Chromatogram of reaction containing ethylenediaminetetraacetic acid, EDTA (F from Figure 1b)

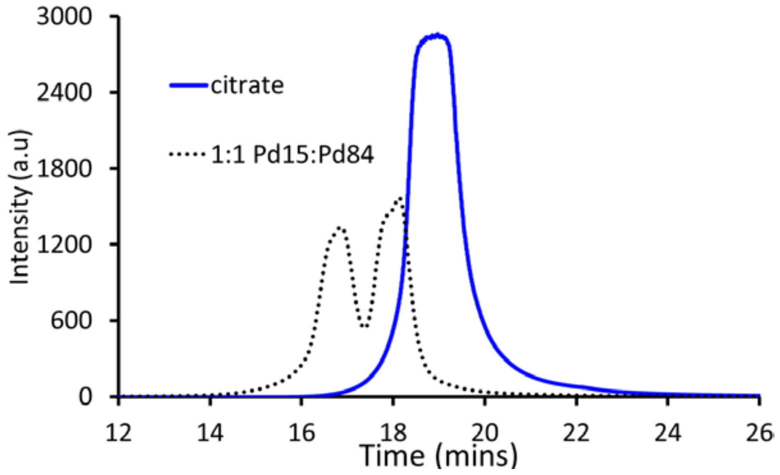


**Figure S8**. Chromatogram of reaction containing sodium citrate (G from Figure 1b)

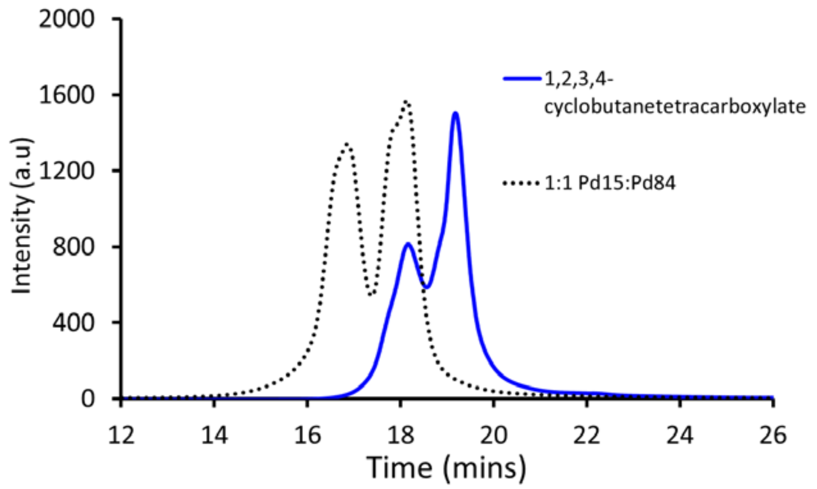


**Figure S9.** Chromatogram of reaction containing 1,2,3,4-cyclobutanetetracarboxylic acid (H from Figure 1b)

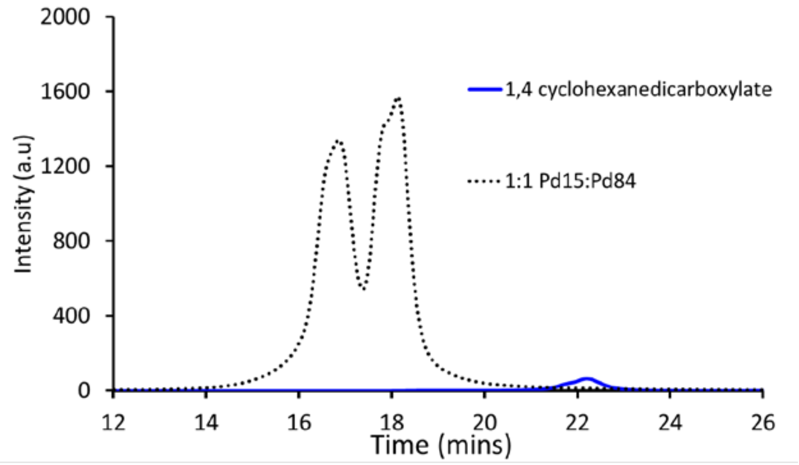


**Figure S10.** Chromatogram of reaction containing 1,4-cyclohexanedicarboxylic acid (I from Figure 1b)

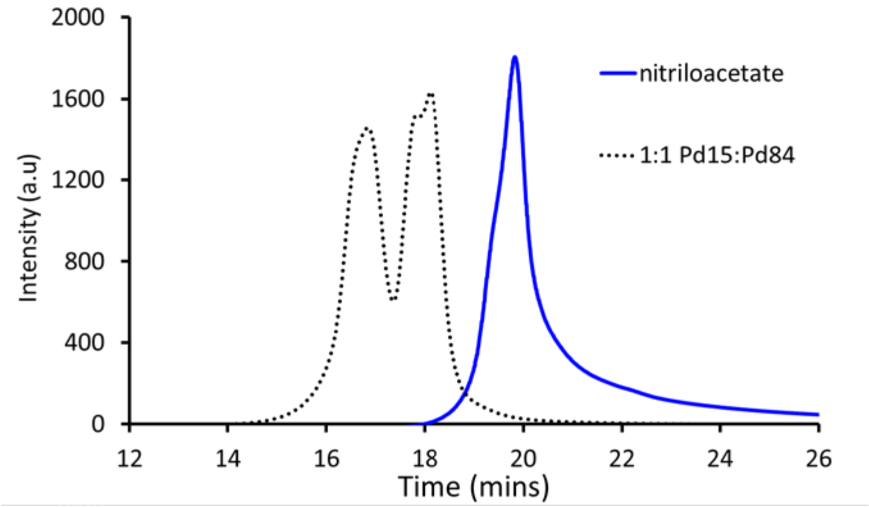


**Figure S11.** Chromatogram of reaction containing nitrilotriacetic acid (J from Figure 1b)

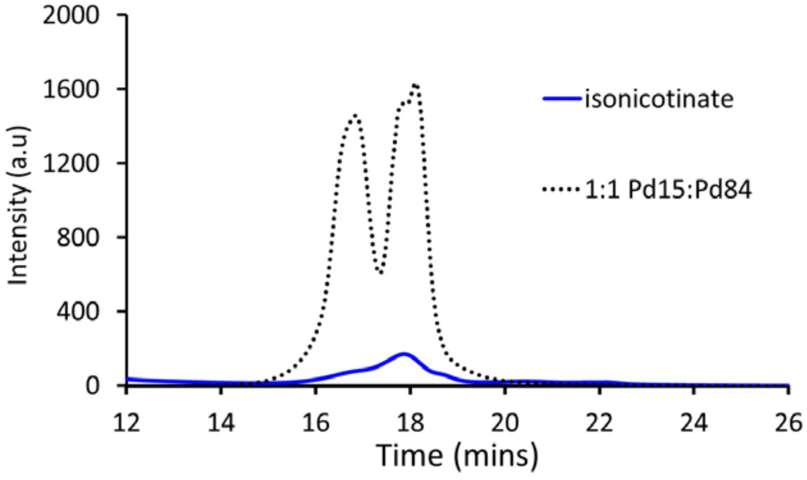


**Figure S12.** Chromatogram of reaction containing isonicotinic acid (K from Figure 1b)

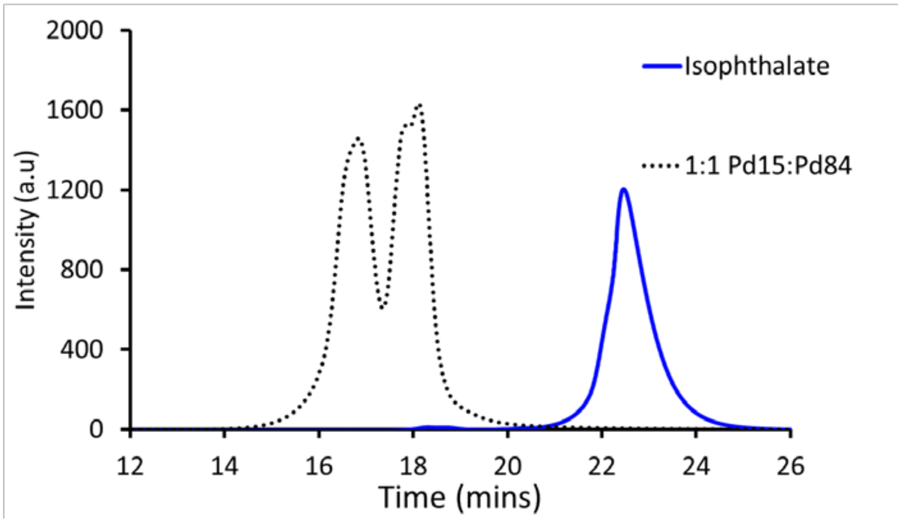


**Figure S13.** Chromatogram of reactions containing isophthalic acid at varying ratios (L from Figure 1b)

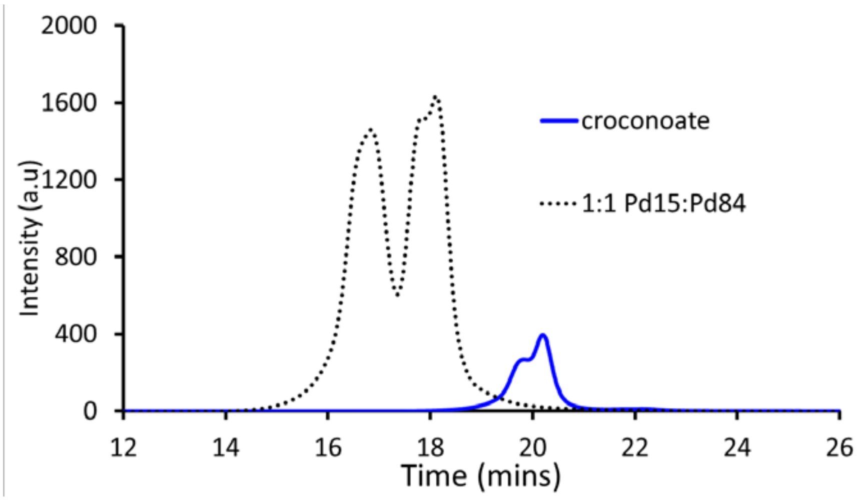


**Figure S14.** Chromatogram of reactions containing croconoic acid at varying ratios (M from Figure 1b)

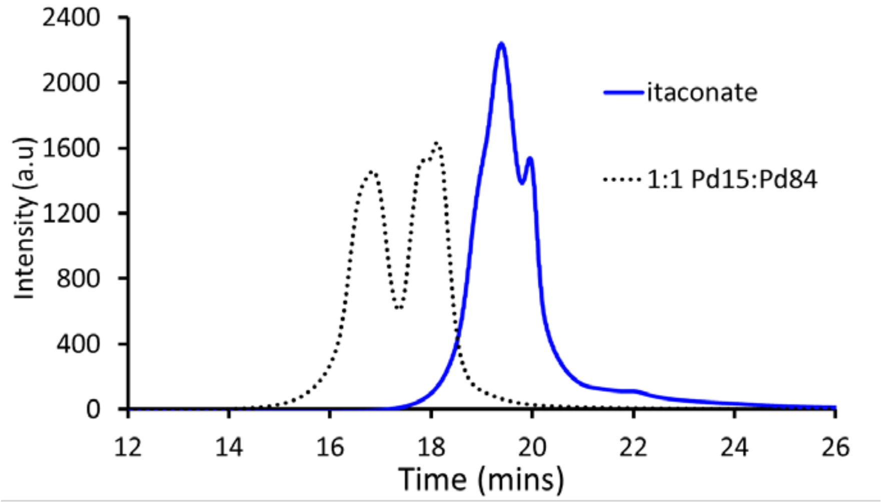


**Figure S15.** Chromatogram of reaction containing itaconic acid (N from Figure 1b)

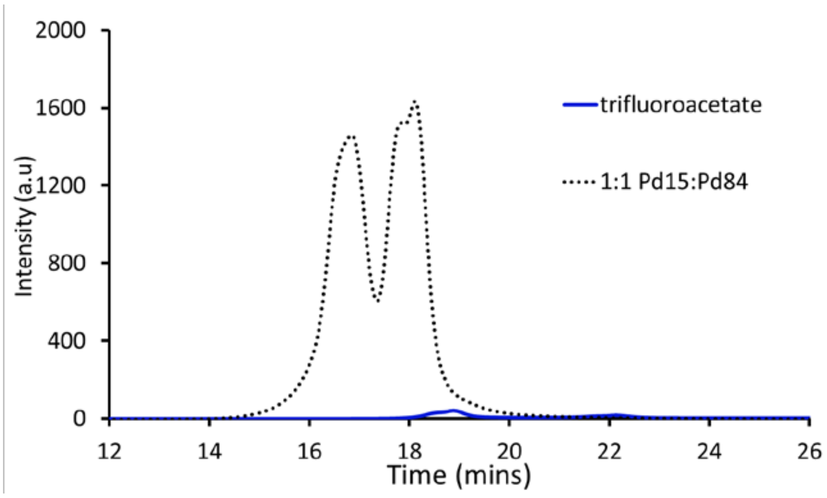


**Figure S16.** Chromatogram of reaction containing sodium trifluoroacetate (P from Figure 1b)

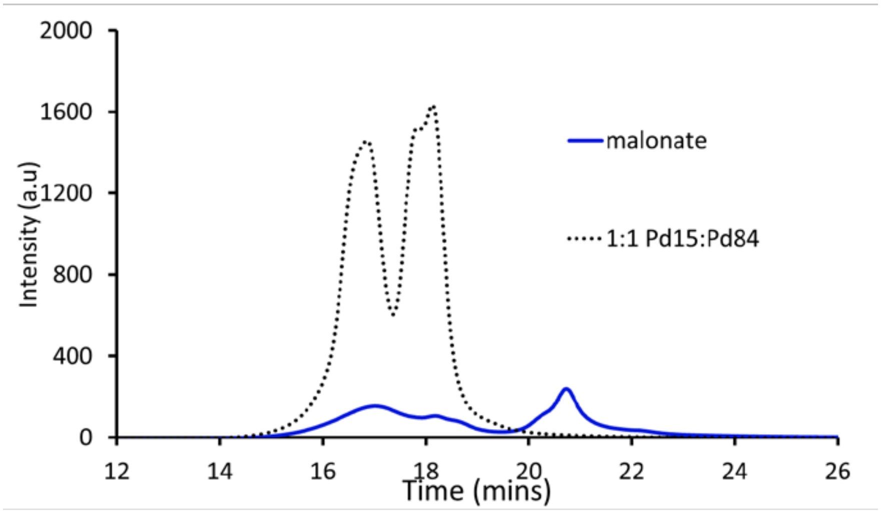


**Figure S17**. Chromatogram of reactions containing sodium malonate at varying ratios (Q from Figure 1b).

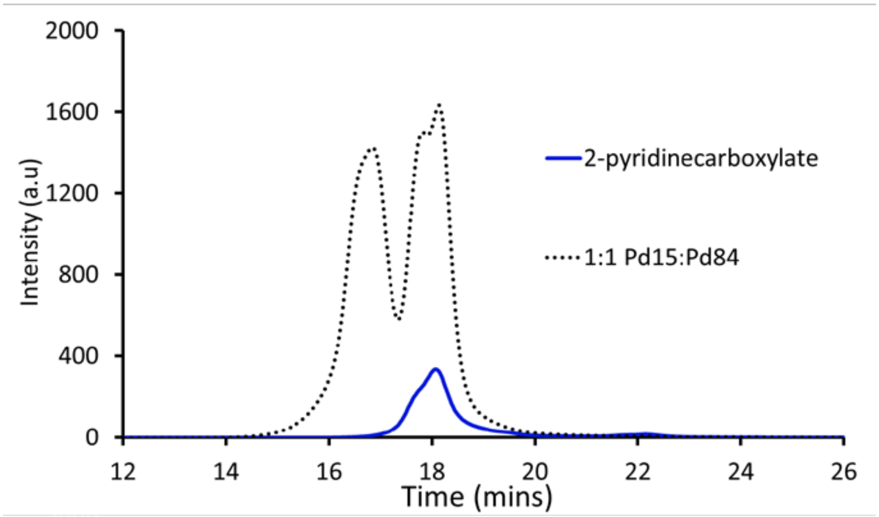


**Figure S18**. Chromatogram of reactions containing 2-pyridinecarboxylic acid at varying ratios (R from Figure 1b)

The screening summary figure (Fig 1b in manuscript) is a representation of the data in the above chromatograms. It is obtained by integration of the SEC chromatograms of the resepective screening reactions (automated in a custom R script)[2] over the "screening range" (Time = 12-17.3 mins). Where products are yielded by the reaction in this size range (corresponding to the size of $\{Pd_{84}\}^{Ac}$, or greater) the integrated intensity is thus large; where little/no products are yielded by the reaction in the desired size range, the integrated intensity is small. The integrated intensities plotted in that figure are normalised to the largest intensity (glycolate reaction, set as 100) for comparison.

**(5) Crystallographic data**

**Table S1. Crystallographic data for $\{Pd_{10}\}^{Prop}$**

| | | |
|---|---|---|
| Identification code | CCDC no. 1484603 | |
| Empirical formula | C42 H69 N3 O28 Pd10 | |
| Formula weight | 2128.00 | |
| Temperature | 150(2) K | |
| Wavelength | 0.71073 Å | |
| Crystal system | Triclinic | |
| Space group | P -1 | |
| Unit cell dimensions | a = 12.7753(3) Å | α = 75.649(2) ° |
| | b = 13.4572(3) Å | β = 89.735(2) ° |
| | c = 20.9469(5) Å | γ = 64.003(2) ° |
| Volume | 3113.35(14) $Å^3$ | |
| Z | 2 | |
| Density (calculated) | 2.270 $Mg/m^3$ | |
| Absorption coefficient | 2.897 $mm^{-1}$ | |
| F(000) | 2052 | |
| Crystal size | 0.154 x 0.119 x 0.042 $mm^3$ | |
| Theta range for data collection | 2.854 to 25.998°. | |
| Index ranges | -15<=h<=15, -16<=k<=16, -25<=l<=25 | |
| Reflections collected | 51315 | |
| Independent reflections | 12216 [R(int) = 0.0521] | |
| Completeness to theta = 25.242° | 99.9 % | |
| Refinement method | Full-matrix least-squares on $F^2$ | |
| Data / restraints / parameters | 12216 / 0 / 763 | |
| Goodness-of-fit on $F^2$ | 1.019 | |
| Final R indices [I>2sigma(I)] | R1 = 0.0295, wR2 = 0.0510 | |
| R indices (all data) | R1 = 0.0480, wR2 = 0.0566 | |
| Extinction coefficient | n/a | |
| Largest diff. peak and hole | 0.719 and -0.774 e.$Å^{-3}$ | |

**Table S2. Crystallographic data for $\{Pd_{72}\}^{Prop}$**

| | | |
|---|---|---|
| Identification code | CCDC no. 1484604 | |
| Empirical formula | C72 H520 Na60 O428 P36 Pd72 | |
| Formula weight | 18391.97 | |
| Temperature | 100(2) K | |
| Wavelength | 0.6889 A | |
| Crystal system | Hexagonal | |
| Space group | P 6 2 2 | |
| Unit cell dimensions | a = 33.2298(5) A | α = 90 ° |
| | b = 33.2298(5) A | β = 90 ° |
| | c = 29.4116(5) A | γ = 120 ° |
| Volume | 28125.8(10) $Å^3$ | |
| Z | 2 | |
| Density (calculated) | 2.172 $Mg/m^3$ | |
| Absorption coefficient | 2.494 $mm^{-1}$ | |
| F(000) | 17776 | |
| Crystal size | 0.200 x 0.060 x 0.030 $mm^3$ | |
| Theta range for data collection | 1.507 to 23.940°. | |
| Index ranges | -39<=h<=39, -39<=k<=39, -34<=l<=34 | |
| Reflections collected | 337378 | |
| Independent reflections | 16040 [R(int) = 0.2110] | |
| Completeness to theta = 25.242° | 99.6 % | |
| Refinement method | Full-matrix least-squares on $F^2$ | |
| Data / restraints / parameters | 16040 / 82 / 533 | |
| Goodness-of-fit on $F^2$ | 1.737 | |
| Final R indices [I>2sigma(I)] | R1 = 0.1727, wR2 = 0.4392 | |
| R indices (all data) | R1 = 0.2180, wR2 = 0.4790 | |
| Absolute structure parameter | 0.47(12) | |
| Extinction coefficient | n/a | |
| Largest diff. peak and hole | 2.48 and -1.30 $e.Å^{-3}$ | |

**Table S2. Crystallographic data for $\{Pd_{84}\}^{Gly}$**

| | | |
|---|---|---|
| Identification code | CCDC no. 1484605 | |
| Empirical formula | C56 H498 Na56 O494 P42 Pd84 | |
| Formula weight | 20604.30 | |
| Temperature | 150(2) K | |
| Wavelength | 0.71073 A | |
| Crystal system | Monoclinic | |
| Space group | C2/m | |
| Unit cell dimensions | a = 57.517(3) Å | α = 90 ° |
| | b = 37.048(3) Å | β = 99.084(4) ° |
| | c = 14.7350(9) Å | γ = 90 ° |
| Volume | 31004(4) $Å^3$ | |
| Z | 2 | |
| Density (calculated) | 2.207 $Mg/m^3$ | |
| Absorption coefficient | 2.625 $mm^{-1}$ | |
| F(000) | 19792 | |
| Crystal size | 0.100 x 0.080 x 0.030 $mm^3$ | |
| Theta range for data collection | 1.434 to 25.702 °. | |
| Index ranges | -69<=h<=70, -45<=k<=45, -15<=l<=17 | |
| Reflections collected | 217157 | |
| Independent reflections | 29934 [R(int) = 0.0804] | |
| Completeness to theta = 25.242° | 100.0 % | |
| Refinement method | Full-matrix least-squares on $F^2$ | |
| Data / restraints / parameters | 29934 / 15 / 1456 | |
| Goodness-of-fit on $F^2$ | 1.149 | |
| Final R indices [I>2sigma(I)] | R1 = 0.0799, wR2 = 0.1995 | |
| R indices (all data) | R1 = 0.1120, wR2 = 0.2361 | |
| Extinction coefficient | n/a | |
| Largest diff. peak and hole | 2.54 and -1.22 e.$Å^{-3}$ | |

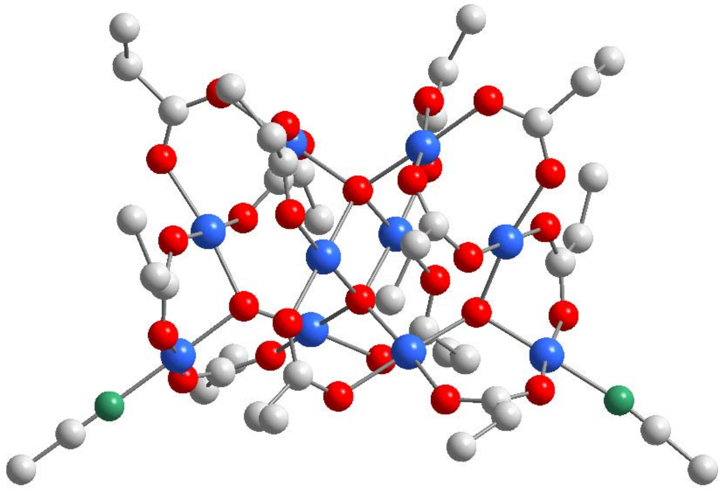

**Figure S19**. Crystal structure of the **{Pd$_{10}$}$^{Prop}$** cluster. Colours: Pd, blue; O, red; N, green; C grey. H omited for clarity.

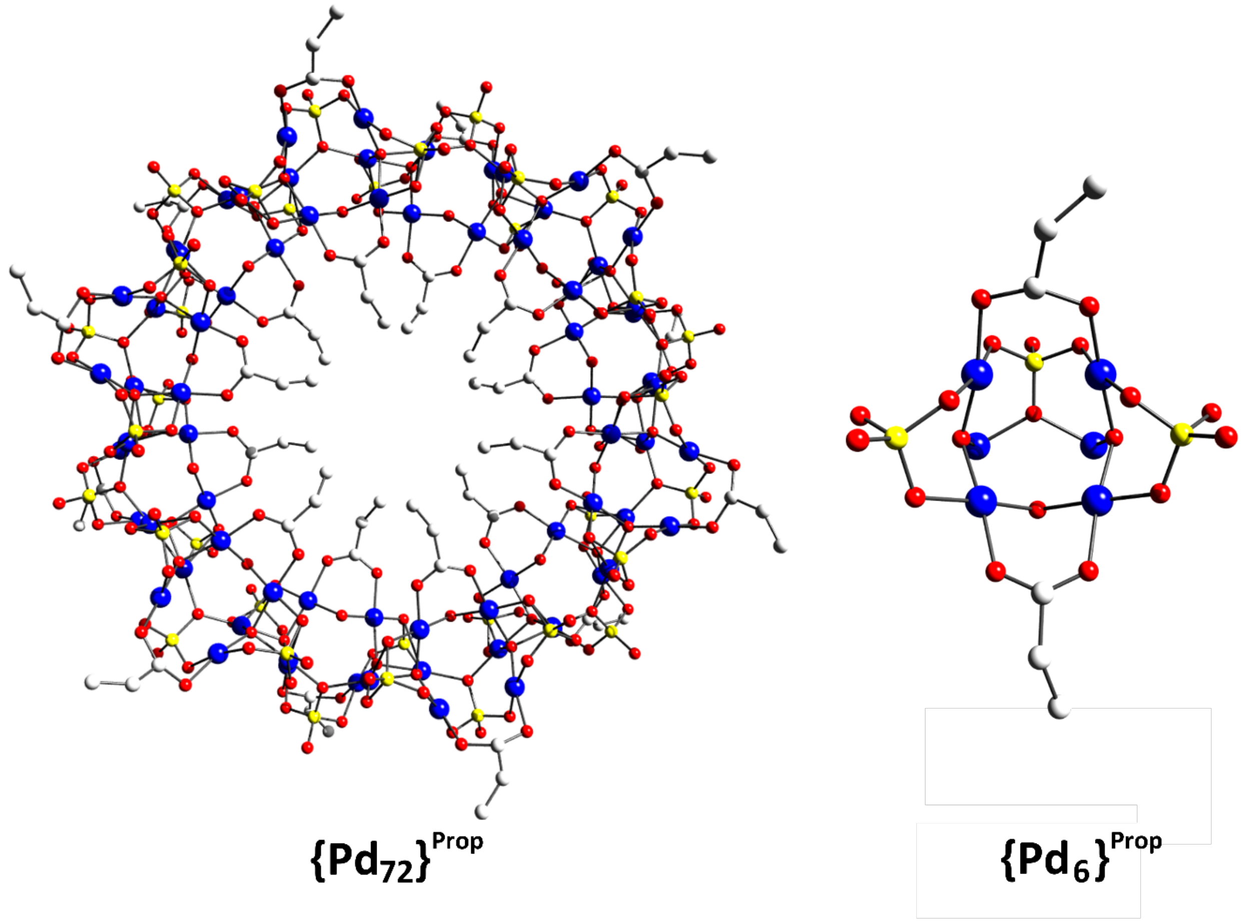


**Figure S20.** Ball and stick structural represenation of {Pd$_{72}$}$^{Prop}$ (left) and the minimal {Pd$_{6}$}$^{Prop}$ building unit (right). Colours: Pd, blue; O, red; P, yellow; C grey. H omited for clarity.

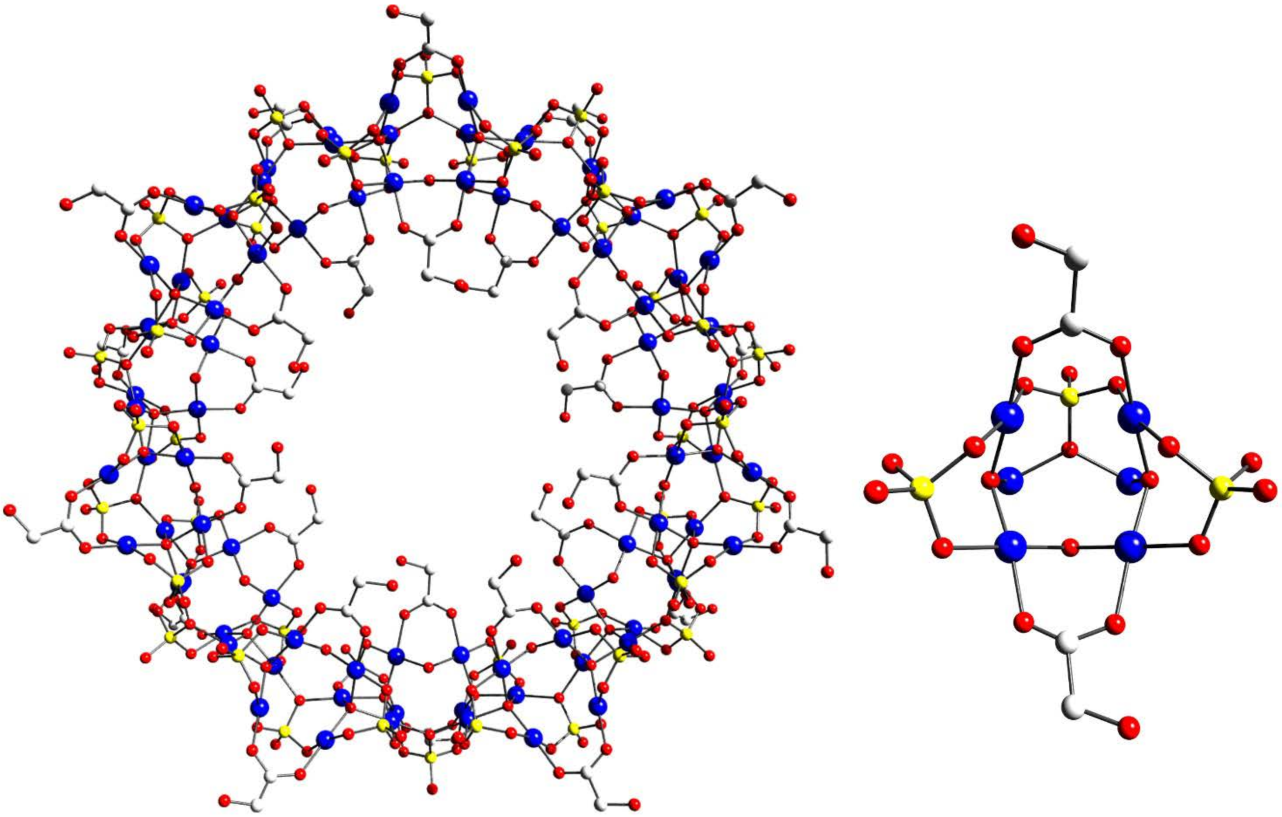

$\{Pd_{84}\}^{Gly}$ $\{Pd_{6}\}^{Gly}$

**Figure S21.** Ball and stick structural represenation of $\{Pd_{84}\}^{Gly}$ (left) and the minimal $\{Pd_{6}\}^{Gly}$ building unit (right). Colours: Pd, blue; O, red; P, yellow; C grey. H omited for clarity.

## (6) $^{31}$P NMR

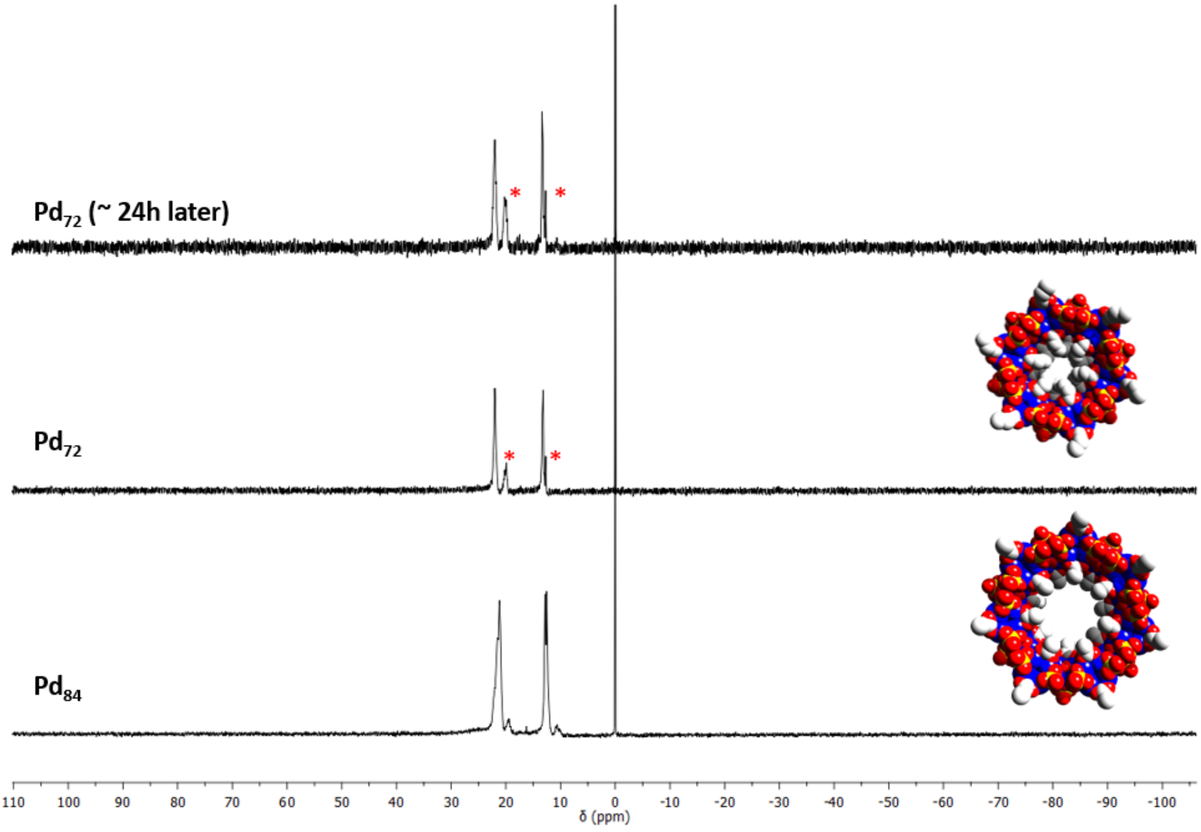


**Figure S22**. $P^{31}$ solution NMR spectra in $D_2O$ for (a) dissolved crystalline sample of $\{Pd_{84}\}^{Ac}$ desalted twice with spin columns, (b) dissoved crystalline samples of $\{Pd_{72}\}^{Prop}$ desalted twice with spin columns and c) the same sample as for b) but left for approximately 24 hours, showing increased amounts of degredation products. Red asterisks indicate the degredation products.

**(7) Degradation of $\{Pd_{72}\}^{Prop}$ in SEC**

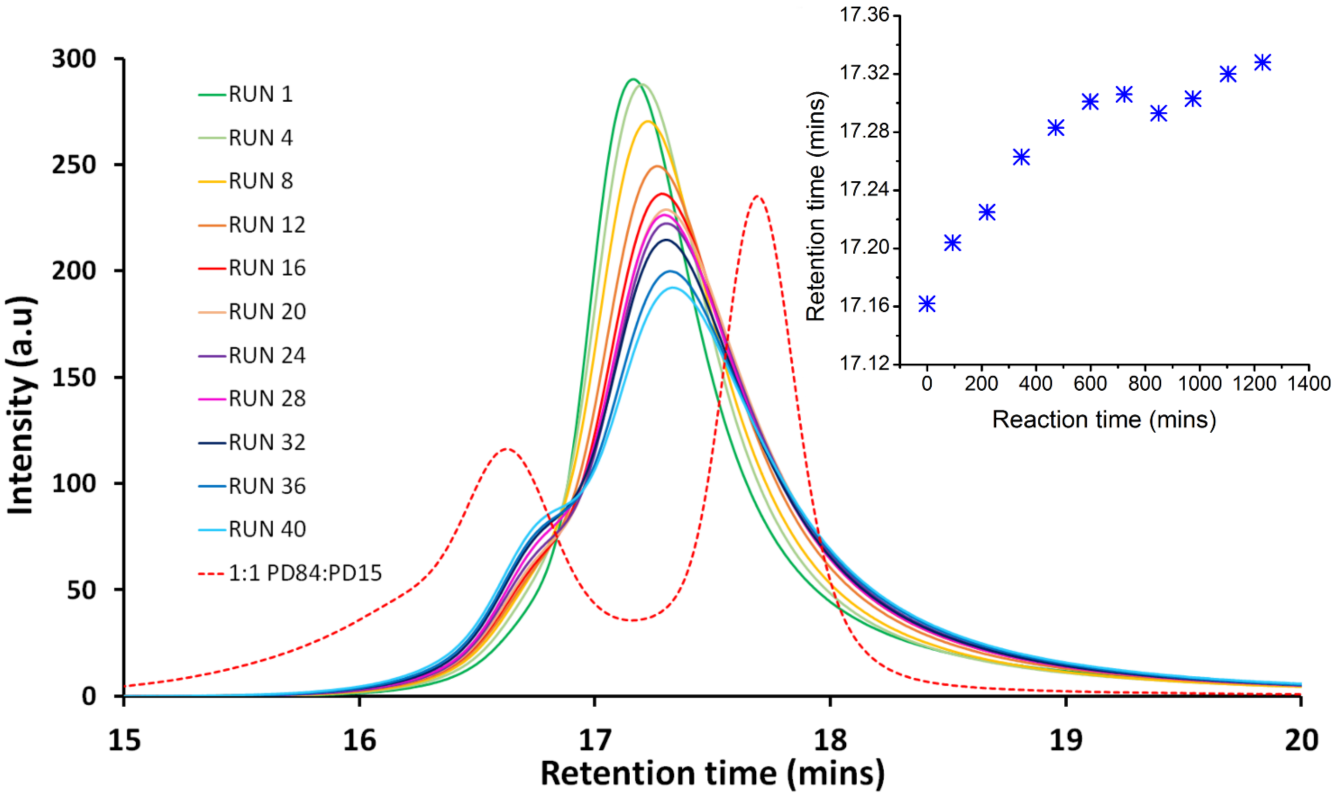


**Figure S23**. SEC traces of a pure crystalline sample of $\{Pd_{72}\}^{Prop}$ dissolved in HPLC $H_2O$ and analysed every 30 minutes for approximately 20 hours. Inset shows the gradual increase in retention time over the analysis period, corresponding to a species of decreasing size.

Degradation of the $\{Pd_{72}\}^{Prop}$ macrocycle was observable in SEC as shown in Figure S23. A pure crystalline sample of $\{Pd_{72}\}^{Prop}$ was dissolved in water and analyzed every 30 mins. Over 20 hours the single peak observed for fresh $\{Pd_{72}\}^{Prop}$ seems to drift to longer retention time (consistent with a smaller species), as a smaller second peak/shoulder appears at shorter retention times (consistent with a larger species).

## (8) ESI-IMS-MS Spectroscopy:

Samples were prepared by dissolving pure crystals of {$Pd_{84}$} in HPLC grade water, at approximately 5 mg/mL; the solutions were desalted twice using MicroBioSpin 6 (TRIS buffer removed by flushing with $NH_4OAc$ solution x 1, then HPLC $H_2O$ x 3), and the resulting solution diluted 50% in HPLC $H_2O$. Samples of {Pd 84-glycolate} were prepared by desalting (as above) a crude reaction solution, and then treated as per 'pure' samples.

Spectra were acquired on a Waters Synapt G2 HDMS instrument in Sensitivity mode (except where otherwise stated), with samples infused into the standard ESI source at 5 µL/min using a Harvard syringe pump. The following parameters were used for acquisition of all spectra (unless otherwise stated): ESI capillary voltage, 2.7 kV; sample cone voltage, 35 V; extraction cone voltage, 4.0 V; source temperature, 80 °C; desolvation temperature, 150 °C; cone gas (N2) flow, 15 L/h; desolvation gas ($N_2$) flow, 750 L/h; source gas flow, 0 mL/min; trap gas flow, 2 mL/min; helium cell gas flow, 180 mL/min; IMS gas flow, 90 mL/ min; IMS DC entrance, 25.0; helium cell DC, 35.0; helium exit, −5; IMS bias, 3.0; IMS DC exit, 0; IMS wave velocity, 1000 m/s; IMS wave height, 40 V. Data were acquired using MassLynx v4.1 and initially processed using DriftScope v2.2. IMS-MS spectra are displayed with a linear intensity scale using the color-coding shown in the accompanying key; no filtering is applied to limit signals/remove noise. To determine drift times (tD) of species of interest in the IMS cell arrival time distribution (ATD), data were extracted from Driftscope/Masslynx, and fitted to Gaussian curves using Fityk v0.9.8 to determine a representative retention times peak center.

Ion mobility calibration data were recorded for equine cytochrome C, oligothymidine,[7] and another DNA strand (d[TTTAGGG]),[8] and fit to their respective helium collision cross sections ($CCS_{He}$) reported in the literature using the approach outlined in reference 3; data were only omitted where peaks were very weak, fitting not possible/ambiguous, or conformations not observed. The resulting calibration curve (Figure S1) was then used to estimate the $CCS_{He}$ of ions of interest from observed drift times.

*We note that during the course of this work we have reported an alternative calibration routine.[9] In this work the purpose of obtaining CCS data is qualitative: to compare the sizes of multiple clusters which have been obtained using the same method, and make structural conclusions based on similarity. As a result, we feel this calibration approach to be sufficient, and to allow comparison with literature values (the majority of metal oxide cluster CCS values in the literature have been reported using the same calibration).[10–14] In biomolecular ESI-IMS-MS it is common to draw quantitative conclusions, based on comparison with putative modelled structures (obtained using software such as Mobcal). We have recently shown[9] that current models (and their respective parameter sets) are not sufficient to draw useful quantitative conclusions about metal oxide cluster structures. Lacking any validated model, here we refrain from employing such an approach.*

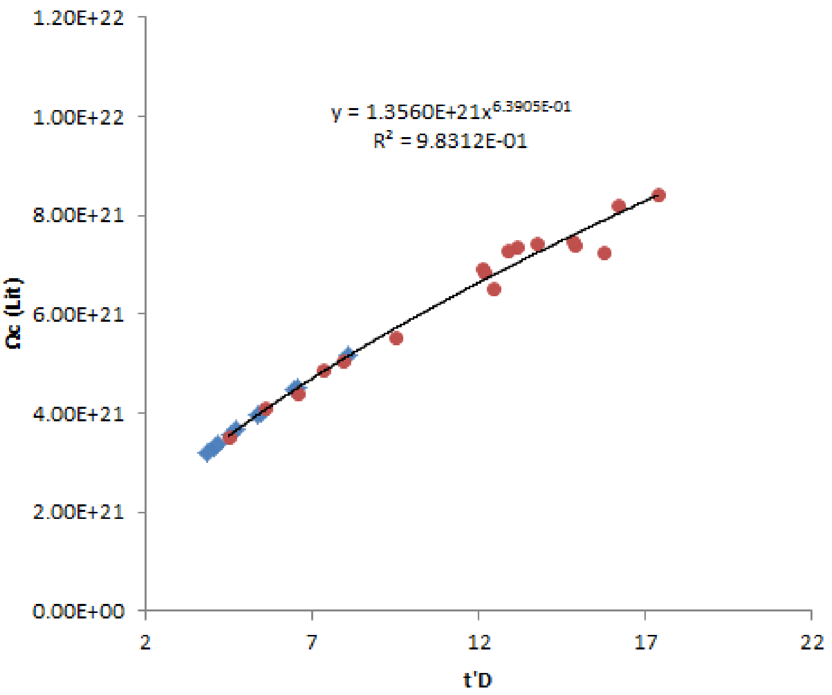


**Figure S24**. Calibration method as per Smith et al, Eur. J Mass Spectrom. 15, 112-130 (2009)[15]

All of the ESI-IMS-MS spectra (Figures S25 – S27) show broad continuous envelopes of related peaks which is quite typical when obtaining ESI-IMS-MS of large POMS. This is attributed to a large mixture of counter ions ($Na^+$ and $H^+$) and solvent molecules ($H_2O$) which are present around the molecules, giving rise to manifold of peaks which can be assigned. For this reason it is possible to assign over a mass range. In addition to this, it is possible that the conditions the macrocycles are exposed to during mass spec may cause the macrocycle to lose some ligands, most likely the outer ligands. The assignments made have accounted for this possibility and we have made several putative assignments for each wheel molecule, taking the value at approximately the centre of the manifold within +/- 2 *m/z* units. This demonstrates the wide possible range of assignments that can be made for these molecules.

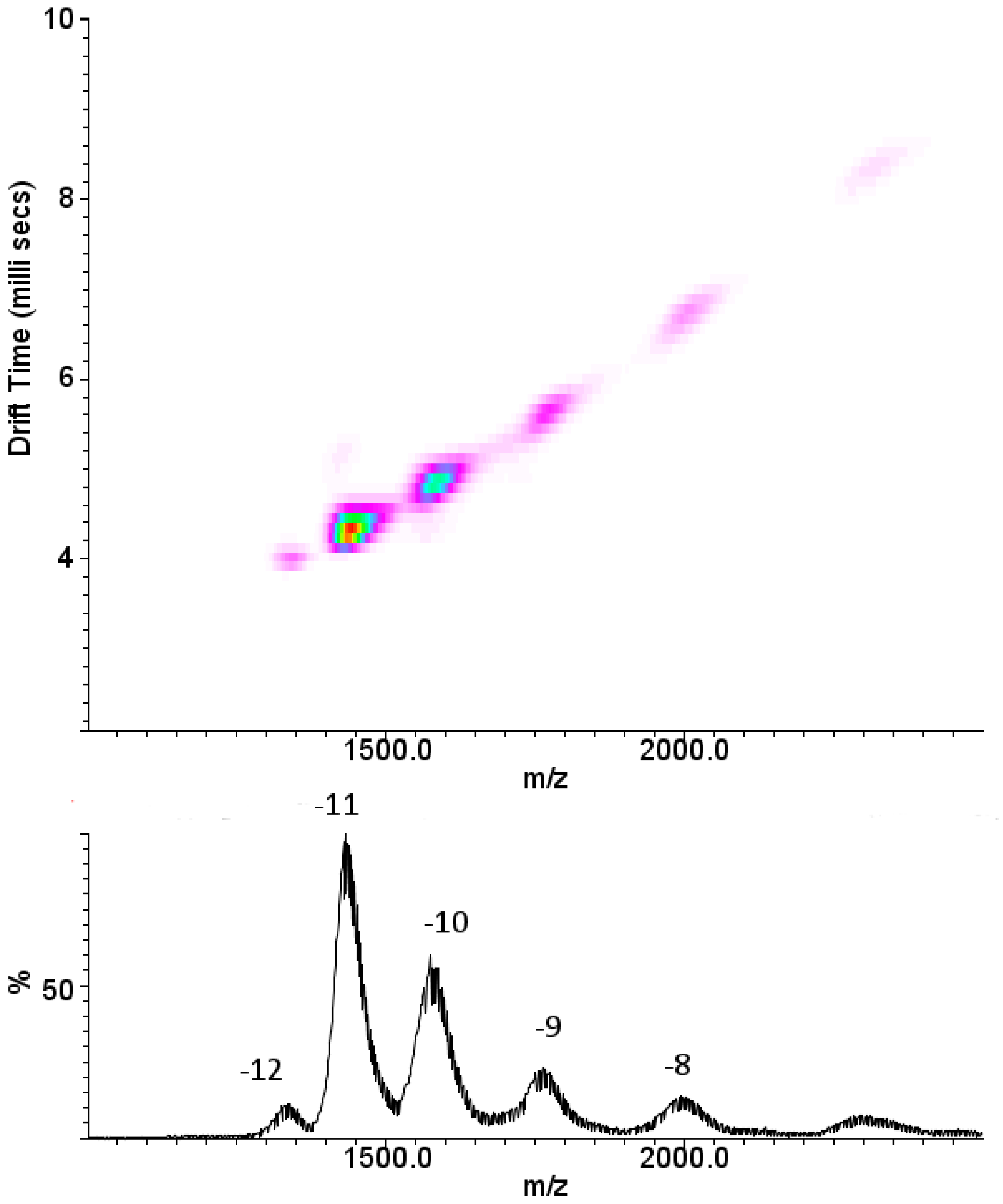


**Figure S25.** ESI-IMS-MS spectrum obtained from prepared **$\{Pd_{84}\}^{Gly}$** mother liquor.

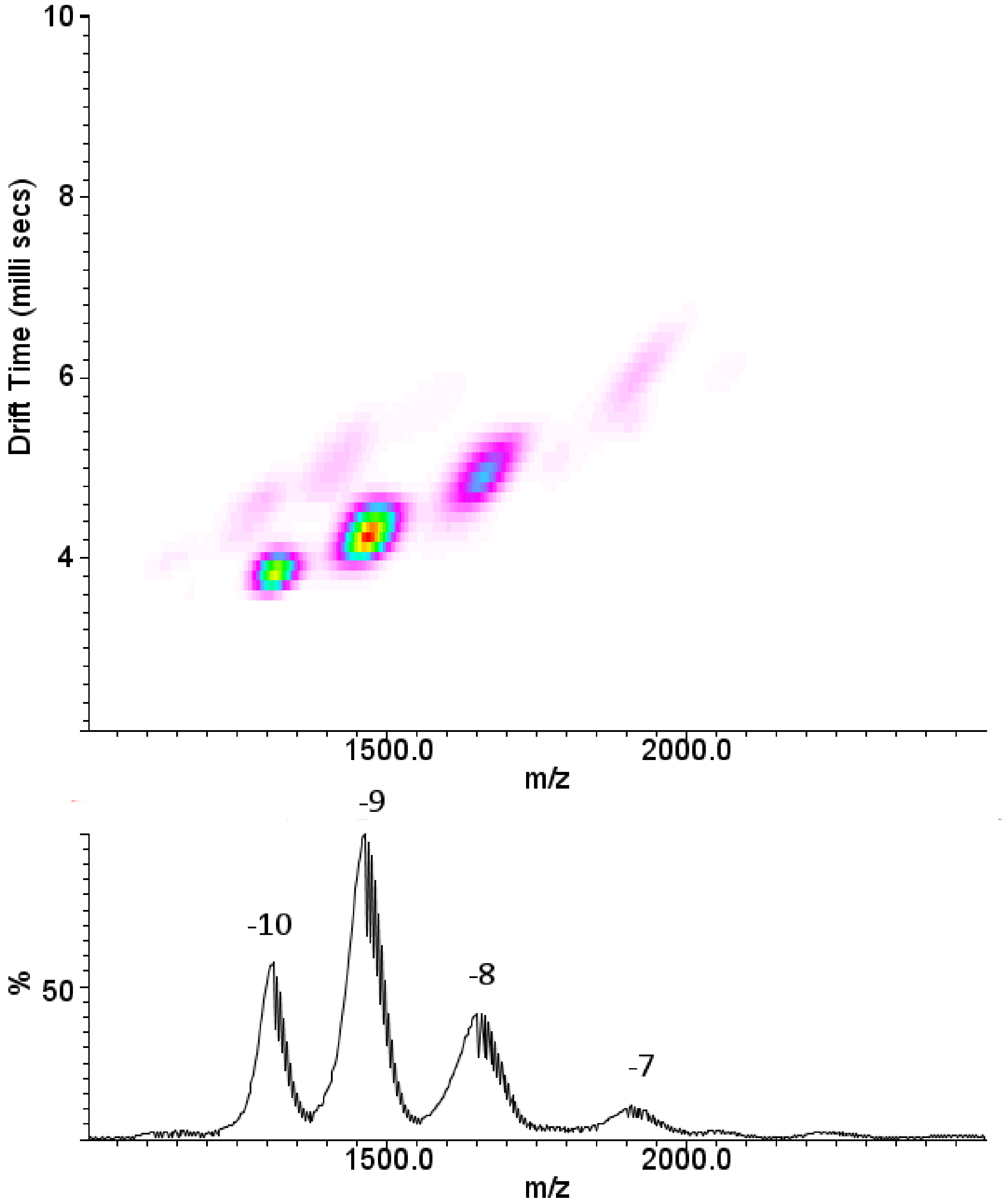


**Figure S26.** ESI-IMS-MS spectrum obtained from prepared **{Pd$_{72}$}$^{Prop}$** crystals.

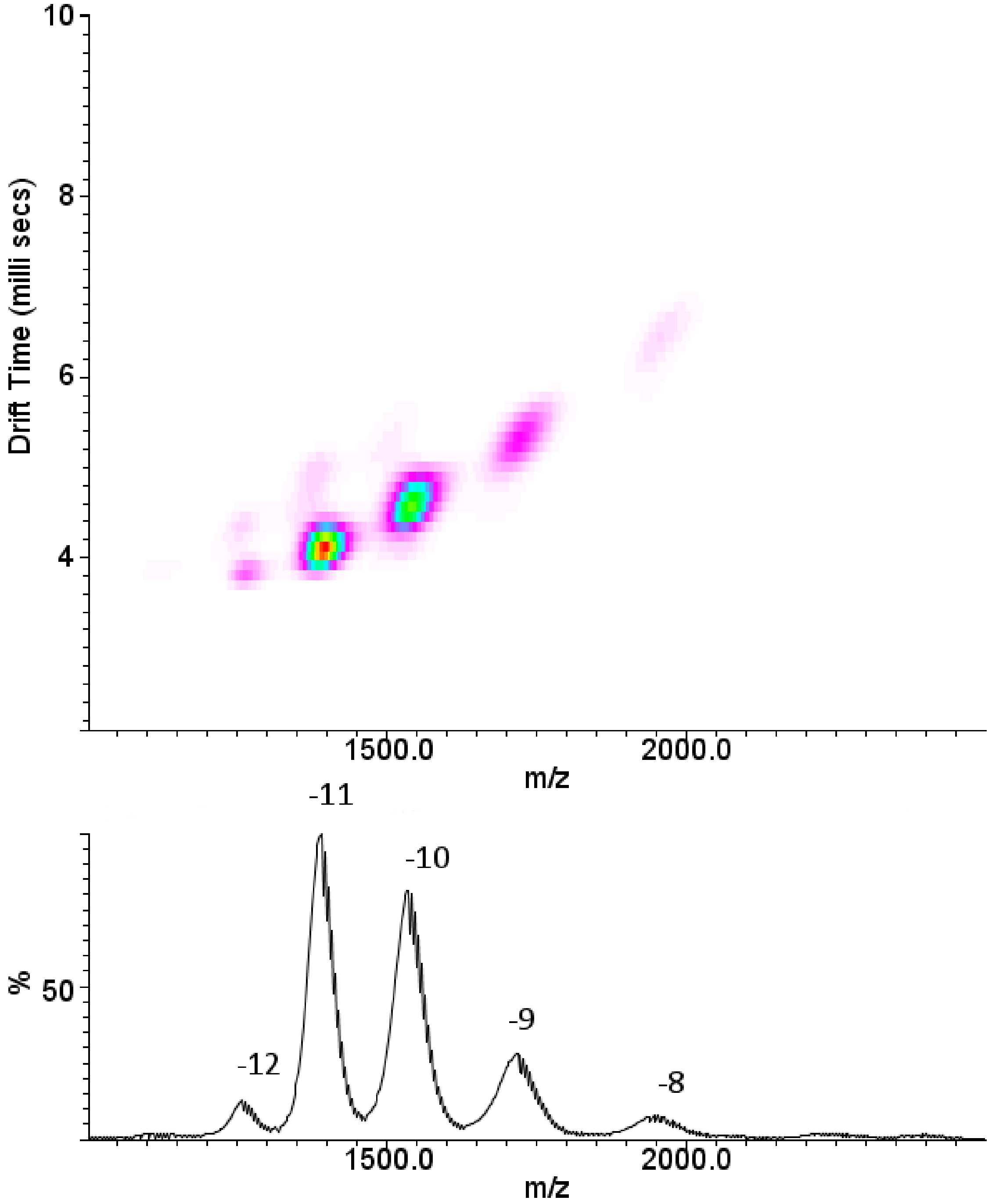


**Figure S27.** ESI-IMS-MS spectrum obtained from prepared **$\{Pd_{84}\}^{Ac}$** crystals.

**Table S4.** A varied selection of possible assignments for the manifold of peaks in the ESI-MS of $\{Pd_{84}\}^{Gly}$. ** indicates an assignment where the wheel remains intact i.e. all 28 glycolate ligands are present.

| m/z observed (centre of the manifold) | Charge state | m/z calculated | Formula (** indicates that the assignment is for a intact wheel i.e. all ligands are present) |
|---|---|---|---|
| 1334 | -12 | 1334.61 | $[Pd_{84}O_{42}(CH_2OHCO_2)_{28}(PO_4)_{42}]$ $(Na)_{10}(H)_{48}(H_2O)_2$** |
| 1334 | -12 | 1334.20 | $[Pd_{84}O_{42}(CH_2OHCO_2)_{26}(PO_4)_{42}]$ $(Na)_{11}(H)_{45}(H2O)_9$ |
| 1334 | -12 | 1334.94 | $[Pd_{84}O_{42}(CH_2OHCO_2)_{28}(PO_4)_{42}]$ $(Na)_{11}(H)_{47}(H2O)_1$** |
| 1435 | -11 | 1434.76 | $[Pd_{84}O_{42}(CH_2OHCO_2)_{28}(PO_4)_{42}]$ $(Na)_1(H)_{58}$** |
| 1435 | -11 | 1436.12 | $Pd_{84}O_{42}(CH_2OHCO_2)_{26}(PO_4)_{42}]$ $(Na)_7(H)_{50}(H2O)_2$ |
| 1435 | -11 | 1435.04 | $[Pd_{84}O_{42}(CH_2OHCO_2)_{25}(PO_4)_{42}]$ $(Na)_5(H)_{51}(H_2O)_8$ |
| 1578 | -10 | 1578.34 | $[Pd_{84}O_{42}(CH_2OHCO_2)_{28}(PO_4)_{42}]$ $(Na)_1(H)_{59}$** |
| 1578 | -10 | 1578.64 | $[Pd_{84}O_{42}(CH_2OHCO_2)_{26}(PO_4)_{42}]$ $(Na)_4(H)_{54}(H_2O)_5$ |
| 1578 | -10 | 1577.24 | $Pd_{84}O_{42}(CH_2OHCO_2)_{27}(PO_4)_{42}(Na)_4(H)_{55}(H_2O)_0$ |
| 1765 | -9 | 1765.16 | $[Pd_{84}O_{42}(CH_2OHCO_2)_{28}(PO_4)_{42}]$ $(Na)_4(H)_{57}(H_2O)_2$** |
| 1765 | -9 | 1765.93 | $[Pd_{84}O_{42}(CH_2OHCO_2)_{27}(PO_4)_{42}]$ $(Na)_7(H)_{53}(H_2O)_3$ |
| 1765 | -9 | 1765.49 | $[Pd_{84}O_{42}(CH2OHCO_2)_{26}(PO_4)_{42}]$ $(Na)_7(H)_{52}(H2O)_7$ |
| 1999 | -8 | 1999.54 | $[Pd_{84}O_{42}(CH_2OHCO_2)_{26}(PO_4)_{42}]$ $(Na)_{11}(H)_{49}(H_2O)_8$ |
| 1999 | -8 | 1999.67 | $[Pd_{84}O_{42}(CH_2OHCO_2)_{27}(PO_4)_{42}]$ $(Na)_{10}(H)_{51}(H_2O)_5$ |
| 1999 | -8 | 1999.44 | $[Pd_{84}O_{42}(CH_2OHCO_2)_{28}(PO_4)_{42}]$ $(Na)_4(H)_{58}(H_2O)_8$ |
| 2300 | -7 | 2300.91 | $[Pd_{84}O_{42}(CH_2OHCO_2)_{28}(PO_4)_{42}]$ $(Na)_9(H)_{54}(H_2O)_8$** |
| 2300 | -7 | 2300.61 | $[Pd_{84}O_{42}(CH_2OHCO_2)_{28}(PO_4)_{42}]$ $(Na)_{13}(H)_{50}(H_2O)_3$** |
| 2300 | -7 | 2300.61 | $[Pd_{84}O_{42}(CH_2OHCO_2)_{27}(PO_4)_{42}]$ $(Na)_{14}(H)_{48}(H_2O)_6$ |

**Table S5.** A varied selection of possible assignments for the manifold of peaks in the ESI-MS of $\{Pd_{72}\}^{Prop}$.

| m/z observed (centre of the manifold) | Charge state | m/z calculated | Formula |
|---|---|---|---|
| 1309 | -10 | 1309.61 | $[Pd_{72}O_{36}(C_2H_5CO_2)_{18}(PO_4)_{36}]$ $(Na)_2(H)_{42}(H_2O)_2$ |
| 1309 | -10 | 1308.21 | $[Pd_{72}O_{36}(C_2H_5CO_2)_{18}(PO_4)_{36}]$ $(Na)_3(H)_{41}(H_2O)_0$ |
| 1309 | -10 | 1309.59 | $[Pd_{72}O_{36}(C_2H_5CO_2)_{17}(PO_4)_{36}]$ $(Na)_7(H)_{36}(H_2O)_0$ |
| 1464 | -9 | 1464.25 | $[Pd_{72}O_{36}(C_2H_5CO_2)_{20}(PO_4)_{36}]$ $(Na)_1(H)_{46}$ |
| 1464 | -9 | 1465.35 | $[Pd_{72}O_{36}(C_2H_5CO_2)_{20}(PO_4)_{36}]$ $(Na)(H)_{47}$ |
| 1464 | -9 | 1463.90 | $[Pd_{72}O_{36}(C_2H_5CO_2)_{19}(PO_4)_{36}]$ $(Na)_3(H)_{43}(H_2O)$ |
| 1655 | -8 | 1655.77 | $[Pd_{72}O_{36}(C_2H_5CO_2)_{20}(PO_4)_{36}]$ $(Na)_2(H)_{46}(H_2O)_2$ |
| 1655 | -8 | 1656.39 | $[Pd_{72}O_{36}(C_2H_5CO_2)_{20}(PO_4)_{36}]$ $(Na)_3(H)_{46}(H_2O)$ |
| 1655 | -8 | 1655.03 | $[Pd_{72}O_{36}(C_2H_5CO_2)_{21}(PO_4)_{36}]$ $(H)_{49}$ |
| 1910 | -7 | 1910.33 | $[Pd_{72}O_{36}(C_2H_5CO_2)_{22}(PO_4)_{36}]$ $(Na)_1(H)_{49}(H_2O)_2$ |
| 1910 | -7 | 1911.03 | $[Pd_{72}O_{36}(C_2H_5CO_2)_{22}(PO_4)_{36}]$ $(Na)_2(H)_{49}(H_2O)$ |
| 1910 | -7 | 1909.88 | $[Pd_{72}O_{36}(C_2H_5CO_2)_{21}(PO_4)_{36}]$ $(Na)_5(H)_{45}(H_2O)_1$ |

**Table S6**. A varied selection of possible assignments for the manifold of peaks in the ESI-MS of $\{Pd_{84}\}^{Ac}$. ** indicates an assignment where the wheel remains intact i.e. all 28 glycolate ligands are present.

| **m/z observed (centre of the manifold)** | **Charge state** | **m/z calculated** | **Formula (** indicates that the assignment is for a intact wheel i.e. all ligands are present)** |
|---|---|---|---|
| 1258 | -12 | 1258.69 | $[Pd_{84}O_{42}(CH_3CO_2)_{22}(PO_4)_{42}]$ $(Na)_7(H)_{45}(H_2O)_0$ |
| 1258 | -12 | 1257.70 | $[Pd_{84}O_{42}(CH_3CO_2)_{24}(PO_4)_{42}]$ $(Na)_1(H)_{53}(H2O)_0$ |
| 1258 | -12 | 1258.20 | $[Pd_{84}O_{42}(CH_3CO_2)_{23}(PO_4)_{42}]$ $(Na)_4(H)_{49}(H_2O)_0$ |
| 1390 | -11 | 1390.86 | $[Pd_{84}O_{42}(CH_3CO_2)_{26}(PO_4)_{42}]$ $(Na)_4(H)_{55}(H2O)_1$ |
| 1390 | -11 | 1390.86 | $[Pd_{84}O_{42}(CH_3CO_2)_{25}(PO_4)_{42}]$ $(Na)_6(H)_{50}(H_2O)_2$ |
| 1390 | -11 | 1390.12 | $[Pd_{84}O_{42}(CH_3CO_2)_{24}(PO_4)_{42}]$ $(Na)_{10}(H)_{45}(H_2O)_0$ |
| 1537 | -10 | 1537.85 | $[Pd_{84}O_{42}(CH_3CO_2)_{28}(PO_4)_{42}]$ $(Na)_3(H)_{57}(H_2O)_0$** |
| 1537 | -10 | 1537.65 | $[Pd_{84}O_{42}(CH_3CO_2)_{27}(PO_4)_{42}]$ $(Na)_4(H)_{55}(H_2O)_2$ |
| 1537 | -10 | 1536.84 | $[Pd_{84}O_{42}(CH_3CO_2)_{26}(PO_4)_{42}]$ $(Na)_8(H)_{50}(H_2O)_0$ |
| 1537 | -10 | 1537.43 | $[Pd_{84}O_{42}(CH_3CO_2)_{25}(PO_4)_{42}]$ $(Na)_{11}(H)_{46}(H_2O)_0$ |
| 1718 | -9 | 1718.61 | $[Pd_{84}O_{42}(CH_3CO_2)_{28}(PO_4)_{42}]$ $(Na)_7(H)_{54}(H_2O)_0$** |
| 1718 | -9 | 1718.83 | $[Pd_{84}O_{42}(CH_3CO_2)_{27}(PO_4)_{42}]$ $(Na)_9(H)_{51}(H_2O)_1$ |
| 1718 | -9 | 1719.04 | $[Pd_{84}O_{42}(CH_3CO_2)_{26}(PO_4)_{42}]$ $(Na)_{11}(H)_{48}(H_2O)_2$ |
| 1948 | -8 | 1948.56 | $[Pd_{84}O_{42}(CH_3CO_2)_{28}(PO_4)_{42}]$ $(Na)_{10}(H)_{52}(H_2O)_3$** |
| 1948 | -8 | 1949.05 | $[Pd_{84}O_{42}(CH_3CO_2)_{28}(PO_4)_{42}]$ $(Na)_{11}(H)_{51}(H_2O)_2$** |
| 1948 | -8 | 1948.04 | $[Pd_{84}O_{42}(CH_3CO_2)_{27}(PO_4)_{42}]$ $(Na)_{15}(H)_{46}(H_2O)_0$ |
| 1948 | -8 | 1947.79 | $[Pd_{84}O_{42}(CH_3CO_2)_{26}(PO_4)_{42}]$ $(Na)_{16}(H)_{44}(H_2O)_2$ |

### (9) Mixed ligand mass spectrometry

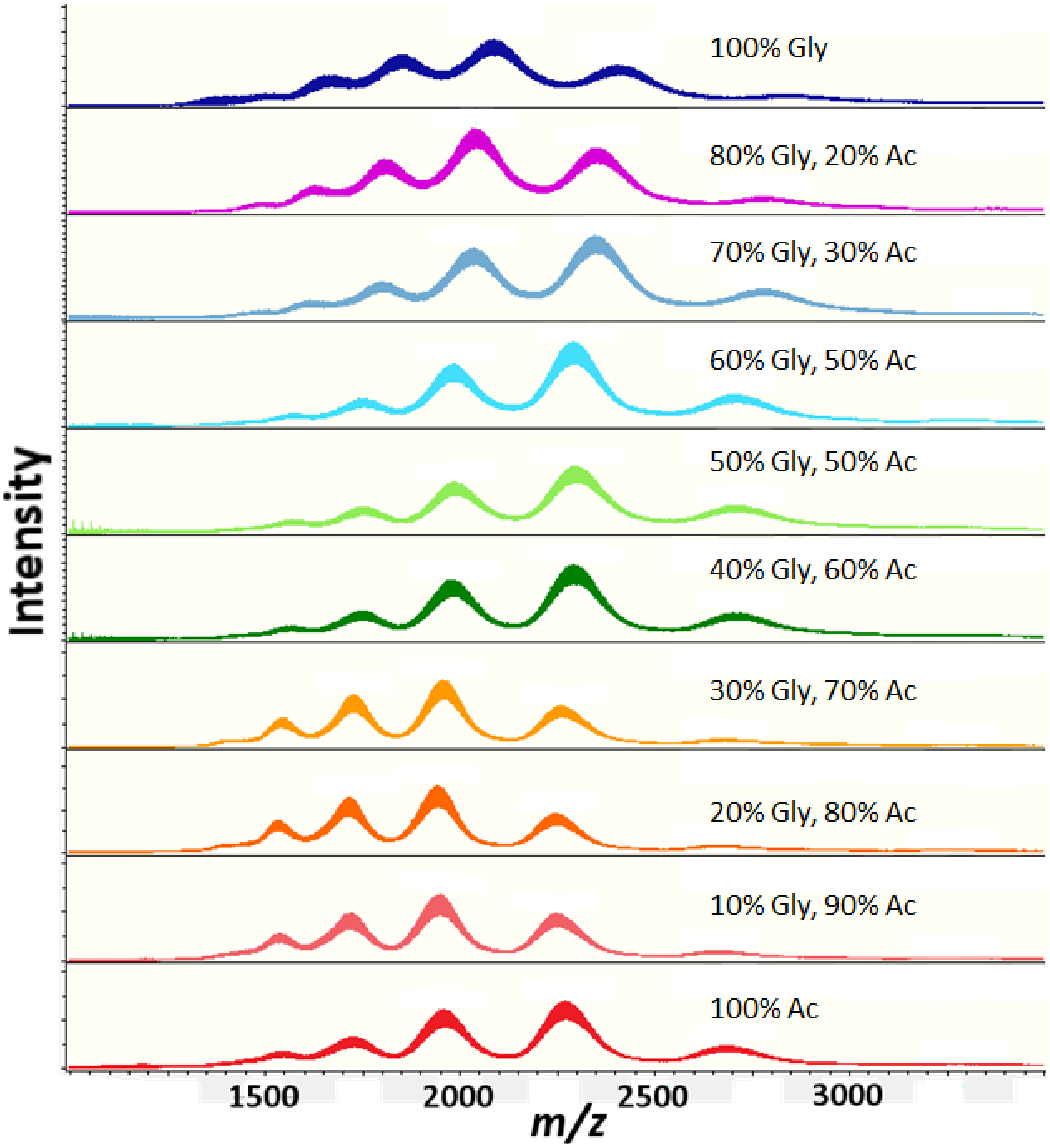


**Figure S28.** Mass spectra of various mixed ligand wheel reactions showing different species can be formed when a ratio of acetate (Ac) and glycolate (Gly) ligands are used.

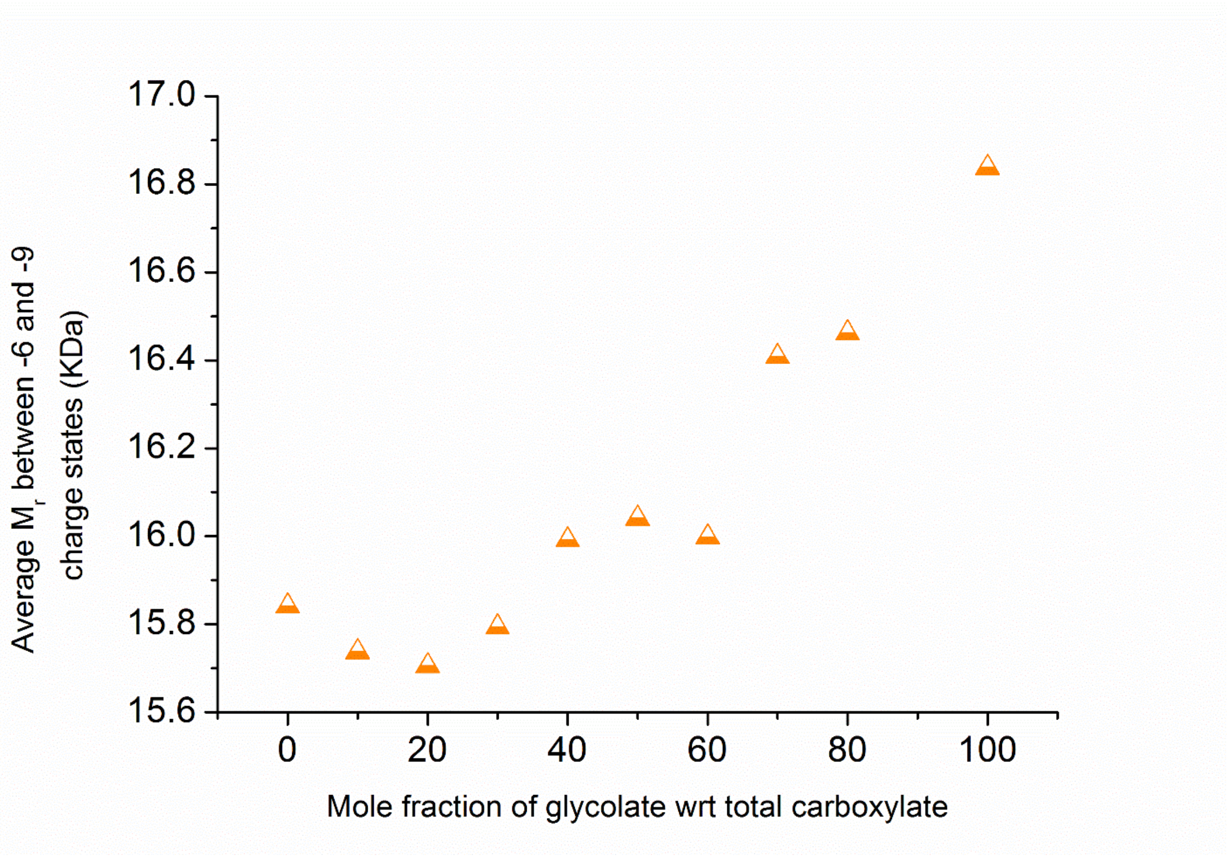


**Figure S29.** Average $M_r$ over the -6 to -9 charge states plotted against mole fraction of glycolate in reaction.

## (10) References


[1] AutoHotkey Foundation LLC, **2013**.

[2] R Core Team, **2013**.

[3] G. M. Sheldrick, *Acta Crystallogr. Sect. A Found. Crystallogr.* **1990**, *46*, 467–473.

[4] G. M. Sheldrick, **2014**.

[5] G. M. Sheldrick, **2014**.

[6] R. Scullion, A. J. Surman, F. Xu, J. S. Mathieson, D.-L. Long, F. Haso, T. Liu, L. Cronin, *Angew. Chem. Int. Ed.* **2014**, *53*, 10032–10037.

[7] C. S. Hoaglund, Y. Liu, a. D. Ellington, M. Pagel, D. E. Clemmer, *J. Am. Chem. Soc.* **1997**, *119*, 9051–9052.

[8] E. S. Baker, S. L. Bernstein, V. Gabelica, E. De Pauw, M. T. Bowers, *Int. J. Mass Spectrom.* **2006**, *253*, 225–237.

[9] A. J. Surman, P. J. Robbins, J. Ujma, Q. Zheng, P. E. Barran, L. Cronin, *J. Am. Chem. Soc.* **2016**, *138*, 3824–3830.

[10] W. Xuan, A. J. Surman, Q. Zheng, D.-L. Long, L. Cronin, *Angew. Chem. Int. Ed.* **2016**, *55*, 1–6.

[11] W. Xuan, A. J. Surman, H. N. Miras, D.-L. Long, L. Cronin, *J. Am. Chem. Soc.* **2014**, *136*, 14114–14120.

[12] P. J. Robbins, A. J. Surman, J. Thiel, D.-L. Long, L. Cronin, *Chem. Comm.* **2013**, *49*, 1909–1911.

[13] A. Macdonell, N. A. B. Johnson, A. J. Surman, L. Cronin, *J. Am. Chem. Soc.* **2015**, *137*, 5662–5665.

[14] G. Izzet, A. Macdonell, C. Rinfray, M. Piot, S. Renaudineau, E. Derat, B. Abécassis, C. Afonso, **2015**, *21*, 19010–19015.

[15] D. Smith, T. W. Knapman, I. Campuzano, R. W. Malham, J. T. Berryman, S. E. Radford, A. E. Ashcroft, *Eur. J. Mass Spectrom.* **2009**, *15*, 113–130.
